# Taxonomic Revision and Conservation Status of *Bartramia* (Bartramiaceae, Bryophyta) in China, with a New Record and an Updated Key

**DOI:** 10.3390/plants15182744

**Published:** 2026-09-08

**Authors:** Xin-Yin Ma, Wen-Zhuan Huang, Yu-Si Liu, Thomas Kiebacher, Sheng-Xia Wang, Yu-Bi Zhou, Yu-Huan Wu

**Affiliations:** 1 College of Life and Environmental Sciences, Hangzhou Normal University, Hangzhou 311121, China; xinyinma@163.com (X.-Y.M.);; 2 Zhejiang Provincial Key Laboratory of Wetland Intelligent Monitoring and Ecological Restoration, Hangzhou Normal University, Hangzhou 311121, China; 3 Zhejiang-Canada Joint Laboratory on Biodiversity and Global Change, Hangzhou Normal University, Hangzhou 311121, China; 4 Stuttgart State Museum of Natural History, Botany Department, Rosenstein 1, 70191 Stuttgart, Germany; 5 Forestry and Grassland Administration, Pingan District, Haidong 810700, China; 6 Qinghai Key Laboratory of Tibetan Medicine Pharmacology and Safety Evaluation, Northwest Institute of Plateau Biology, Chinese Academy of Sciences, Xining 810008, China

**Keywords:** bryoflora, East Asia, morphology, mosses, taxonomy

## Abstract

The genus *Bartramia*, commonly known as “Apple Moss,” is characterized by its globose to subglobose capsules. However, the species diversity and distribution of the genus in China remain incompletely known due to the lack of a comprehensive taxonomic revision. In this study, we conducted a taxonomic revision of this genus in China, based on recent field collections and herbarium specimens. In total, 120 specimens from twelve provinces in China were examined. *Bartramia deciduifolia* is newly reported for China, where it occurs in Qinghai, Sichuan, Xizang, and Yunnan. Seven species are recognized in China: *B. brevifolia*, *B. deciduifolia*, *B. halleriana*, *B. ithyphylla*, *B. laevisphaera*, *B. pomiformis*, and *B. subulata*. Among them, *B. laevisphaera* is proposed as an endangered [EN B2ab(ii, iii)] species due to its small population and habitat, while the other six species are Least Concern (LC). Detailed morphological descriptions, color illustrations, and distributional information are provided, together with an updated identification key to the Chinese species.

## 1. Introduction

*Bartramia* Hedw. is a taxonomically difficult genus of Bartramiaceae Schwägr., with approximately 70 accepted species worldwide [1]. The genus is distributed mainly in temperate regions and in montane areas of the tropics and subtropics [2,3]. It is characterized by erect, often tomentose plants, leaves usually differentiated into a sheath and limb, papillose to mammillose upper laminal cells, and globose to subglobose capsules that are usually furrowed when dry [2,3,4,5,6]. Although there have been many taxonomic studies on this genus (e.g., [2,3,4]), species delimitation in *Bartramia* remains difficult due to its phenotypic plasticity, overlapping gametophytic characters, and variable sporophytic traits [2,3,4,5,7]. In China, the species diversity and distribution of the genus remain incompletely understood because available collections have not been comprehensively revised and some regions remain insufficiently sampled [6,8].

The earliest records of *Bartramia* from China were *B. halleriana* Hedw. and *B. pomiformis* Hedw., both reported by Bescherelle [9] from Yunnan Province, southwestern China. Subsequently, Brotherus [10] reported *B. ithyphylla* Brid. and *B. subulata* Bruch & Schimp. from Yunnan Province. Two additional species, *B. leptodonta* Wilson and *B. subpellucida* Mitt., were reported from Yunnan by Zang and Li [11], although both are now treated as synonyms of *B. brevifolia* Brid. by Fransén [2]. In addition, *Anacolia laevisphaera* (Taylor) Flowers, recorded from Taiwan Province by Iwatsuki and Sharp [12], was later treated as a synonym of *B. laevisphaera* (Taylor) Müll. Hal. [13]. Li [14] reported *B. fragilifolia* Müll. Hal. from Sichuan Province; however, Redfearn et al. [15] considered this record doubtful, and the species was not included in the subsequent treatment in the Moss Flora of China by Li et al. [6]. Thus, a total of six *Bartramia* species were recognized in China before the present study. Nevertheless, a comprehensive revision of the available Chinese collections remained necessary, and the species diversity and distribution of *Bartramia* in China therefore required reassessment.

China harbors the world’s greatest diversity of bryophytes, with more than 3411 recognized taxa [16,17]. In recent years, reassessing the conservation status of these taxa has become increasingly important because of growing anthropogenic pressures, habitat degradation, and the availability of new distribution data [18,19]. However, due to the lack of distribution data and the relatively few bryologists who participate in conservation assessments, only 186 bryophyte species have been classified as threatened in China [20,21]. Notably, no species of *Bartramia* are included on the Threatened Species List [21]. Assessing the conservation status of *Bartramia* species is therefore essential for ensuring their long-term persistence in China and informing effective bryophyte conservation strategies.

To clarify the species diversity and the conservation status of *Bartramia* in China, we conducted extensive fieldwork and re-examined herbarium specimens. The objectives of this study were (1) to verify the species diversity and distribution of *Bartramia* in China; (2) to provide morphological descriptions, distributional information, and illustrations for the Chinese *Bartramia* species; (3) to assess the conservation status of Chinese *Bartramia* species; and (4) to provide an identification key to the species of *Bartramia* in China.

## 2. Results

Seven species of *Bartramia* are recognized in China, including *B. brevifolia*, *B. deciduifolia*, *B. halleriana, B. ithyphylla*, *B. laevisphaera*, *B. pomiformis*, and *B. subulata*. Among them, *B. deciduifolia* is newly recorded for China. Additionally, *B. laevisphaera* is proposed as an endangered [EN B2ab(ii, iii)] species due to its small population and habitat, while the other six species are Least Concern (LC).

### 2.1. Taxonomy

#### 2.1.1. *Bartramia brevifolia* Brid., Bryol. Univ. 2: 737. 1827. (Figure 1 and Figure 2)

Chinese name: 毛叶珠藓 (Máo Yè Zhū Xiǎn)

Type: ECUADOR. Loja, “Americae Aequinoctialis prope Loxam,” *A. von Humboldt & A. Bonpland s.n.* (lectotype: G, designated by Fransén [4], not seen).

Description: Plants medium-sized, excluding sporophytes, 1.8–2.6 cm tall, densely tomentose. Stems simple or sparsely branched, 0.2–0.5 mm in diameter, with a central strand occupying 20–30% of stem diameter. Leaves 5.1–5.4 mm long, erect-appressed when dry, erect-spreading when moist, differentiated into a hyaline sheathing base and a green subulate limb. Sheathing base 0.8–1.1 mm long, 0.4–0.7 mm wide at shoulders, cells smooth, hyaline, linear to elongate-rectangular, unistratose. Costa distinct in the sheathing base, in transverse section with two layers of guide cells. Costa rough abaxially in the limb, in transverse section with two layers of guide cells, well-developed stereid bands, and differentiated dorsal and ventral epidermal cells. Limb 4.0–4.1 mm long, 0.2–0.3 mm wide at base; middle laminal cells short-rectangular to linear, 6.5–31.5 × 3.1–7.5 μm, with high mammillae. Margins more or less revolute, slightly recurved at shoulders, serrulate.

Synoicous. Antheridia clavate, 0.5–0.7 mm long, borne among archegonia; Perichaetial leaves similarly long as vegetative leaves. Setae 1.0–1.9 cm long. Capsules inclined, asymmetric, ovoid, 2.1–3.0 mm long, 1.3–2.0 mm in diameter; exothecial cells irregularly polygonal, 55.5–120.0 μm long and 31.0–97.5 μm wide, thick-walled. Stomata restricted to the capsule base, superficial. Peristome double; exostome teeth yellowish-brown, transversely trabeculate, finely papillose, 210–350 μm long; endostome consisting of a basal membrane and well-developed, finely papillose segments. Spores 16–30 μm in diameter, subglobose, coarsely papillose.

Distribution: In China, this species has been recorded from Sichuan, Xizang, and Yunnan (as *Bartramia leptodonta* or *B. subpellucida* [11,22,23,24]). According to our results, this species is mainly distributed in southwestern China, while the records from Anhui Province (as *Bartramia subpellucida* [25,26]) are doubtful and require further confirmation.

**Figure 1:**
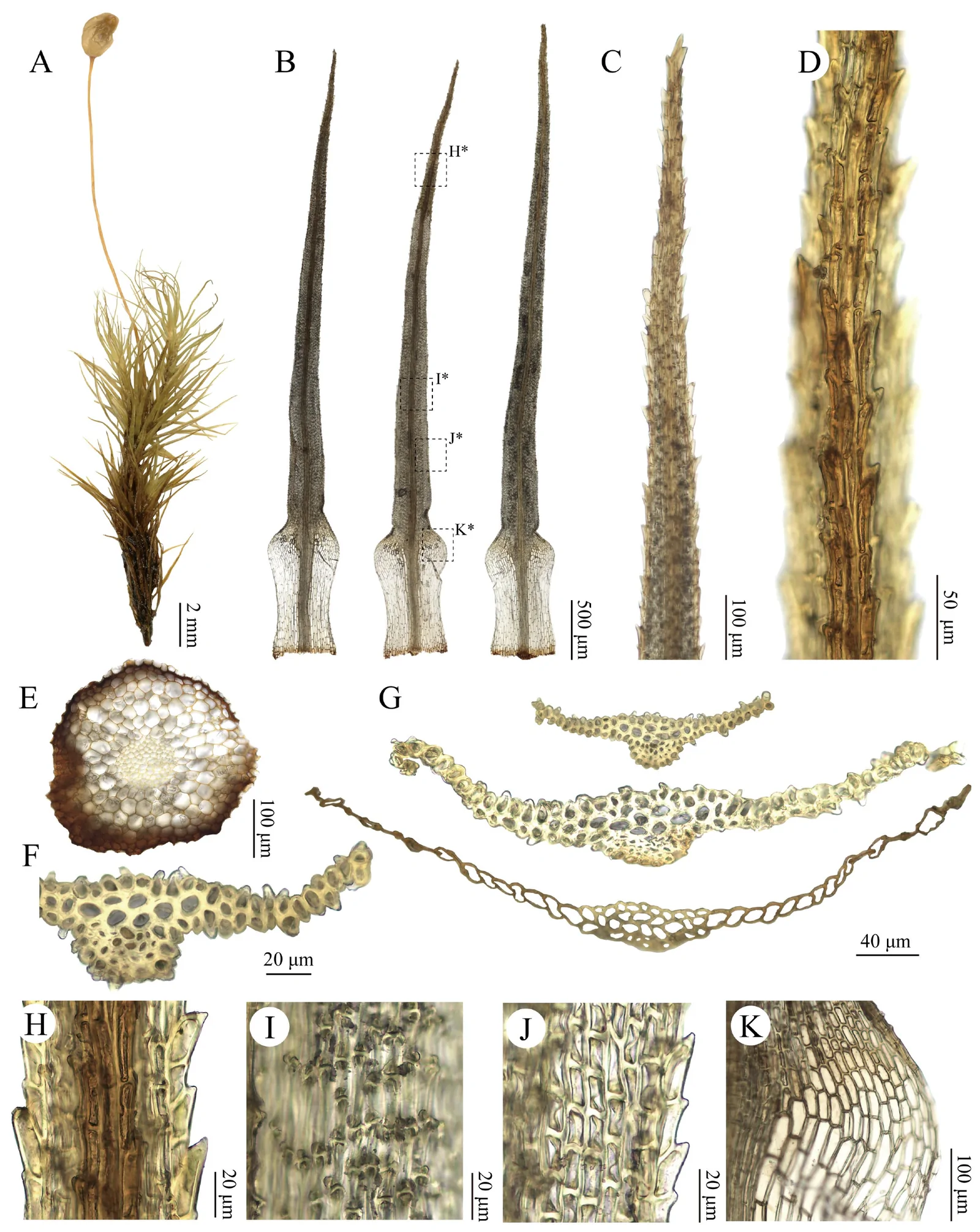
*Bartramia brevifolia* Brid. (**A**) Moist plants with mature sporophytes. (**B**) Leaves. (**C**) Leaf apex. (**D**) Abaxial costal cells of the limb. (**E**) Transverse section of stem. (**F**) Transverse section of the mid-limb. (**G**) Transverse sections of leaves. (**H**) Upper limb cells. (**I**) Basal limb cells. (**J**) Shoulder cells. (**K**) Sheathing-base cells. (**H**), (**I**), (**J**), and (**K**) are enlarged details of H*, I*, J*, and K*, respectively. All from *L.-S. Wang et al. 05-25162 (KUN)*.

**Figure 2:**
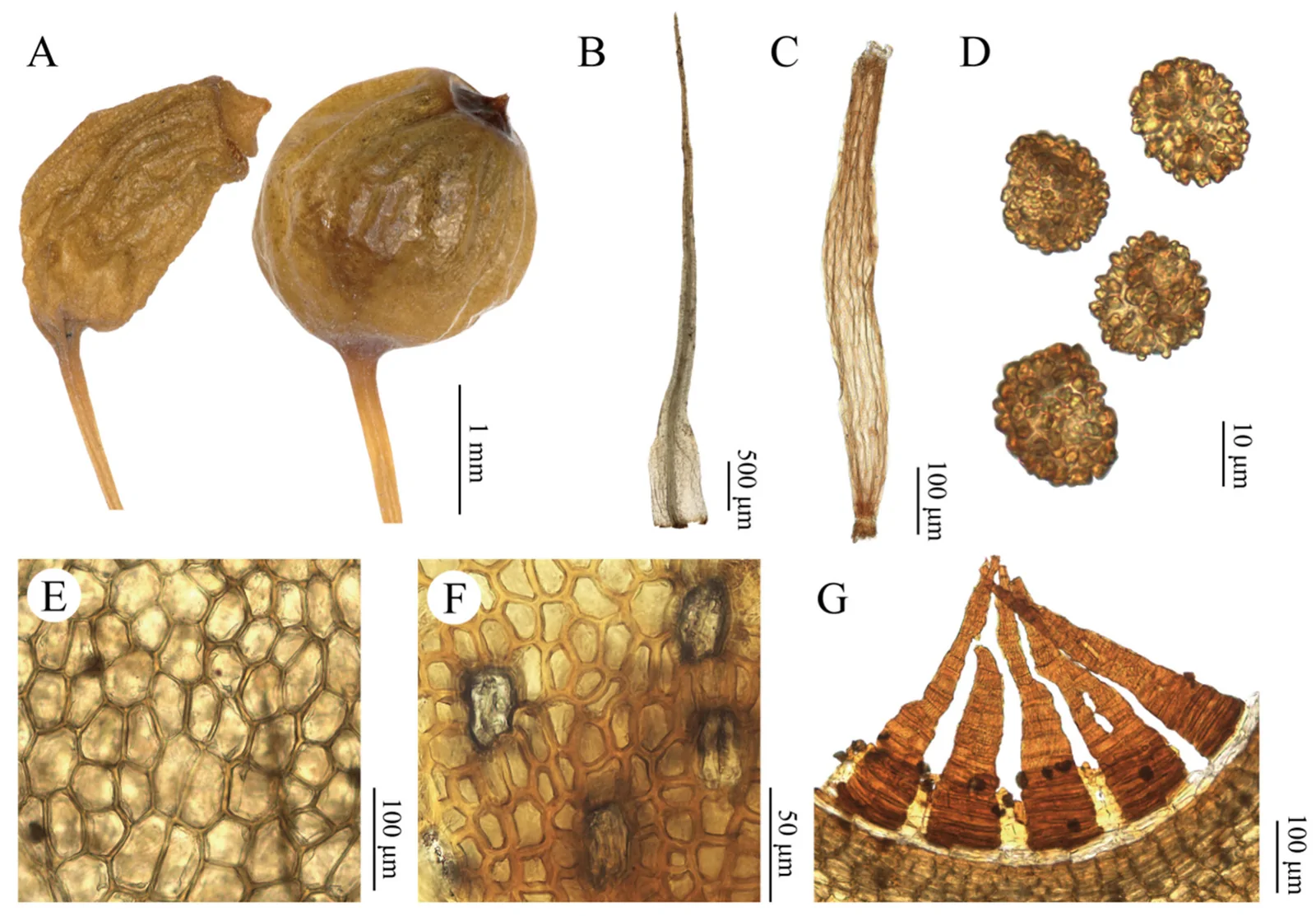
*Bartramia brevifolia* Brid. (**A**) Capsules, dry (**left**) and moist (**right**). (**B**) Perichaetial leaves. (**C**) Antheridia. (**D**) Spores. (**E**) Exothecial cells. (**F**) Stomata. (**G**) Peristome. All from *L.-S. Wang et al. 05-25162* (KUN).

**Figure 3:**
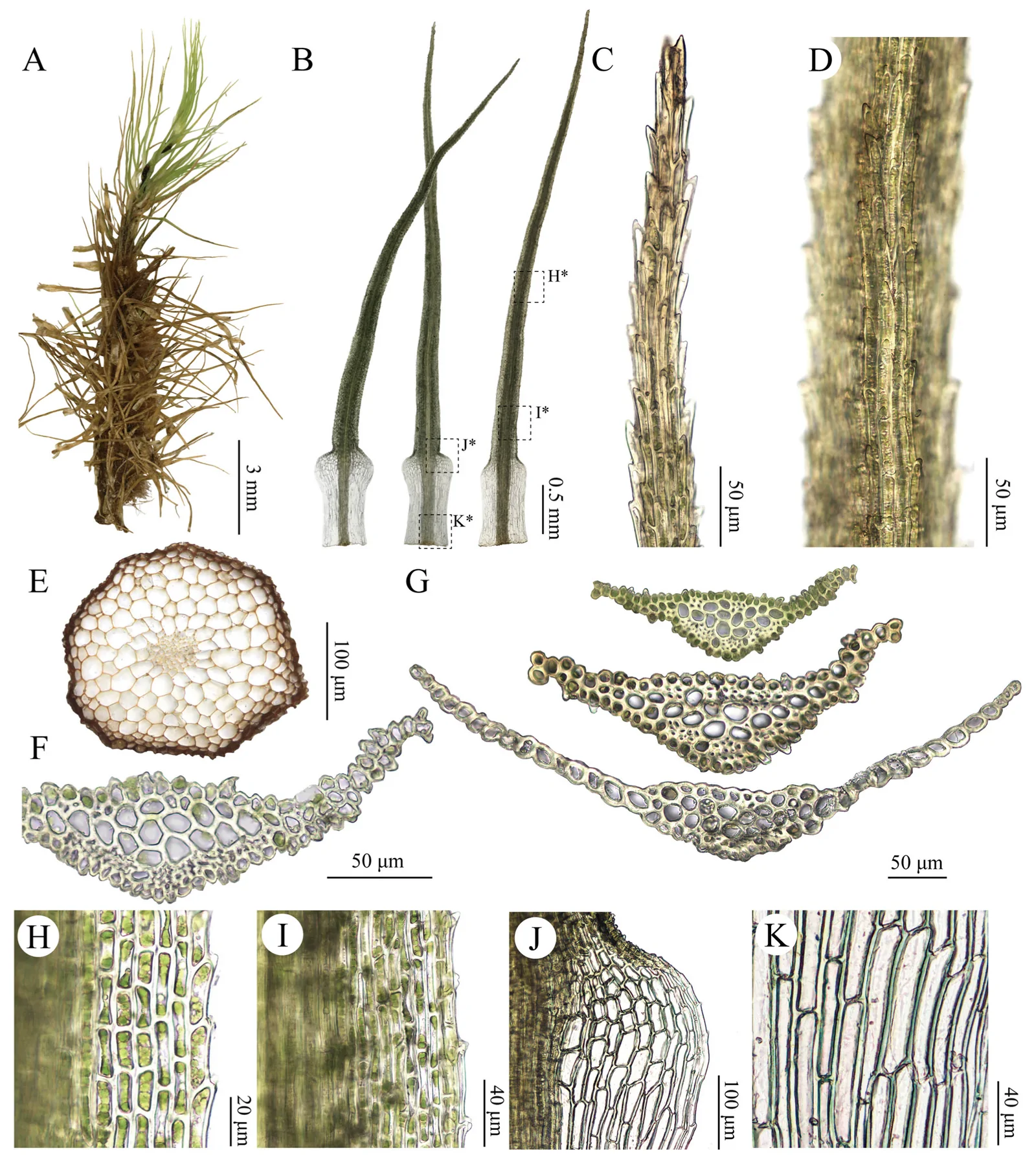
*Bartramia deciduifolia* Broth. & Yasuda. (**A**) Moist plants. (**B**) Leaves. (**C**) Leaf apex. (**D**) Abaxial costal cells of the limb. (**E**) Transverse section of stem. (**F**) Transverse section of the mid-limb. (**G**) Transverse sections of leaves. (**H**) Upper limb cells. (**I**) Basal limb cells. (**J**) Shoulder cells. (**K**) Sheathing-base cells. (**H**), (**I**), (**J**), and (**K**) are enlarged details of H*, I*, J*, and K*, respectively. All from *W.-Z. Huang & F.-Y. Zhang 20241012-78* (HTC).

**Figure 7:**
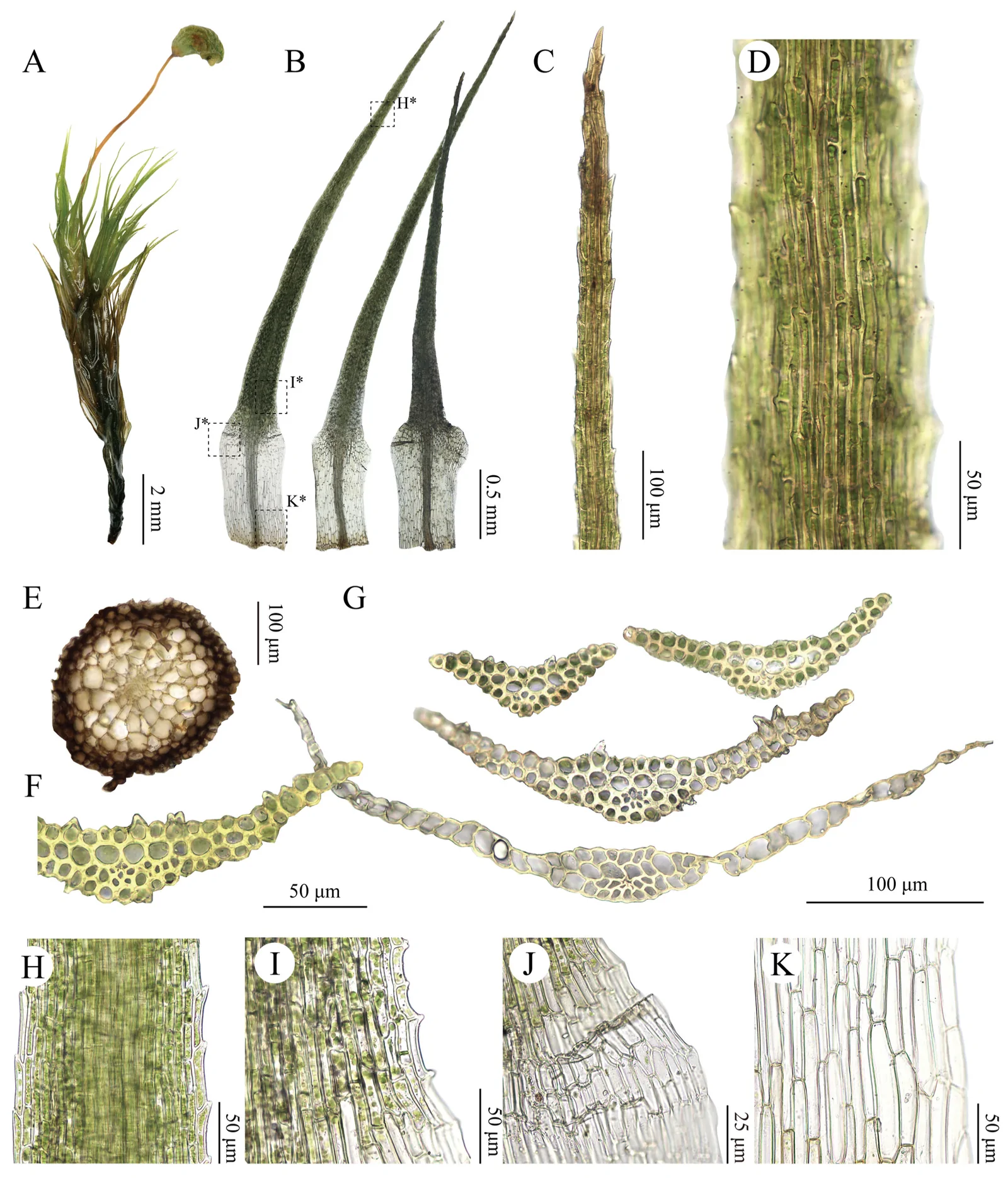
*Bartramia ithyphylla* Brid. (**A**) Moist plant with a mature sporophyte. (**B**) Leaves. (**C**) Leaf apex. (**D**) Abaxial costal cells of the limb. (**E**) Transverse section of stem. (**F**) Transverse section of the mid-limb. (**G**) Transverse sections of leaves. (**H**) Upper limb cells. (**I**) Basal limb cells. (**J**) Shoulder cells. (**K**) Sheathing-base cells. (**H**), (**I**), (**J**), and (**K**) are enlarged details of H*, I*, J*, and K*, respectively. All from *W.-Z. Huang et al. 2025072736* (HTC).

Notes: *Bartramia brevifolia* is morphologically similar to *B. ithyphylla*; however, the two species can be distinguished by the following characters: (1) the leaf limb margins are usually revolute in *B. brevifolia* [2,4,27] (Figure 1B,G), whereas they are plane or only slightly recurved in *B. ithyphylla* [2,6,27] (Figure 7B,G); (2) in transverse section, the costa has two layers of guide cells in *B. brevifolia* [2] (Figure 1F,G), whereas it has a single layer of guide cells in *B. ithyphylla* [2] (Figure 7F,G); (3) the limb cells have high, distinct mammillae in *B. brevifolia* [2,27] (Figure 1F,G,I), whereas those of *B. ithyphylla* bear low mammillae [2,27] (Figure 7F,G,I).

#### 2.1.2. *Bartramia deciduifolia* [Original Spelling: *deciduaefolia*] Broth. & Yasuda, Bot. Mag. (Tokyo) 29(337): 23. 1915. (Figure 3 and Figure 4)

Chinese name: 落叶珠藓 (Luò Yè Zhū Xiǎn)

Type: JAPAN. Honshu, Prov. Kotsuke, Mt Akagi, 19 September 1913, *K. Tsunoda 98* (holotype: H; isotype: BM; not seen).

Description: Plants excluding sporophytes 1.0–2.0 cm tall, forming dense tufts. Stems 0.2–0.4 mm in diameter, branched, with a central strand occupying 10–20% of the stem diameter, lower portions not tomentose. Leaves 5.4–6.1 mm long, easily deciduous, differentiated into a hyaline sheathing base and a green subulate limb. Sheathing base 0.9–1.0 mm long, 0.5–0.6 mm wide at shoulders, hyaline, unistratose; cells smooth, hyaline, linear to elongate-rectangular, 54–189 × 5.5–27.0 μm. Costa in the sheathing base diffuse; in transverse section with two layers of large guide cells, small stereid bands, and differentiated dorsal and ventral epidermal cells. Limb 4.5–5.0 mm long, ca. 0.3 mm wide at base; marginal border unistratose, ca. 4 cells wide; limb cells narrowly rectangular to linear, 16.5–53.5 × 3.0–12.5 μm, with low mammillae. Costa in the limb diffuse, rough abaxially; in transverse section with two layers of large guide cells, stereid group small, and differentiated dorsal and ventral epidermal cells. Margins plane, slightly recurved at the shoulders, serrulate.

Synoicous. Antheridia clavate, borne among archegonia; archegonia flask-shaped, 0.6–0.8 mm long, with a slender neck and swollen venter. Perichaetial leaves not longer than vegetative leaves. Setae 11.9–12.4 mm long. Capsules inclined, asymmetric, ovoid, 1.4–2.0 mm long, 1.0–1.7 mm in diameter; exothecial cells irregularly polygonal, 23.5–60.0 μm long and 19.0–71.5 μm wide, thick-walled. Stomata restricted to the capsule base, superficial. Peristome single; exostome teeth yellowish-brown, complete, transversely trabeculate, finely papillose; endostome absent. Spores 28.5–35.5 μm, reniform, yellowish-brown, moderately papillose.

Distribution. New to China: Qinghai, Sichuan, Xizang, and Yunnan.

Notes: *Bartramia deciduifolia* was described from Japan by Brotherus and Yasuda [28] and later treated as a synonym of *B. ithyphylla* by Ochi [29] and Noguchi [30]. Fransén [2] subsequently reinstated *B. deciduifolia* as a distinct species in his taxonomic revision of the extra-Neotropical species of *Bartramia* sect. *Vaginella*. In China, the species may previously have been overlooked or misidentified as *B. ithyphylla*, particularly when the deciduous nature of the leaves was not apparent. Detailed diagnostic differences between the two species are provided under *B. ithyphylla*.

#### 2.1.3. *Bartramia halleriana* Hedw., Sp. Musc. Frond.: 164. 1801. (Figure 5 and Figure 6)

Chinese name: 亮叶珠藓 (Liàng Yè Zhū Xiǎn)

Type: locality not indicated on the specimen; third specimen from the left on the Hedwig–Schwägrichen sheet (lectotype: G [G00040042], designated by Ederra [31], not seen).

Description: Plants excluding sporophytes 1.8–3.6 cm tall, densely tomentose. Stems ca. 0.3–0.4 mm in diameter, branched, with a central strand occupying ca. 15% of stem diameter, lower portions densely tomentose. Leaves 6.9–7.4 mm long, slightly flexuose but not strongly curled when dry, patent when moist, linear-lanceolate, gradually narrowed from base to apex, rather indistinctly differentiated into a sheathing base and limb. Leaf base erect, green, 0.5–0.6 mm wide; lamina usually unistratose, occasionally partly bistratose. Costa in the sheathing base distinct; in transverse section with two layers of guide cells and a well-developed stereid group. Costa long-excurrent and rough abaxially in the limb; in transverse section with two to three layers of guide cells and a well-developed stereid group. Margins revolute and serrulate. Upper laminal cells rectangular to narrowly rectangular, 6.5–25.0 × 2.5–5.5 μm, papillose; median cells rectangular to narrowly rectangular, 6.0–23.5 × 2.5–7 μm; basal cells linear to elongate-rectangular, 12.5–72.5 × 2.5–5.5 μm, shorter toward the margins; alar cells short-rectangular to quadrate.

Synoicous. Antheridia clavate, 0.6–1.0 mm long. Archegonia flask-shaped, 0.6–1.0 mm long, with a slender neck and swollen venter. Perichaetial leaves not longer than vegetative leaves, ovate-lanceolate, 3.4 mm long. Setae 0.4–2.7 mm long. Capsules inclined, ovoid, 2.1–2.3 mm long, 1.1–1.5 mm in diameter, furrowed when dry; exothecial cells short-rectangular to rectangular, 41.5–100.0 μm long and 33.0–50.5 μm wide, thick-walled. Stomata restricted to the capsule base, superficial. Peristome double; exostome teeth yellowish-brown, transversely trabeculate, finely papillose; endostome with a basal membrane and well-developed segments, finely papillose. Spores spherical, 15–24 μm in diameter, brownish to dark brown, with papillae or verrucae aggregated into small clusters.

Distribution. China: Anhui [26], Chongqing, Fujian, Guangdong [32], Gansu [33], Guizhou, Hebei, Heilongjiang, Hubei [34], Hunan, Inner Mongolia, Jiangsu, Jiangxi, Jilin, Liaoning, Qinghai (new record), Shaanxi, Sichuan, Taiwan [35], Xinjiang, Xizang [36], Yunnan [11], and Zhejiang [15,22,23,24].

**Figure 5:**
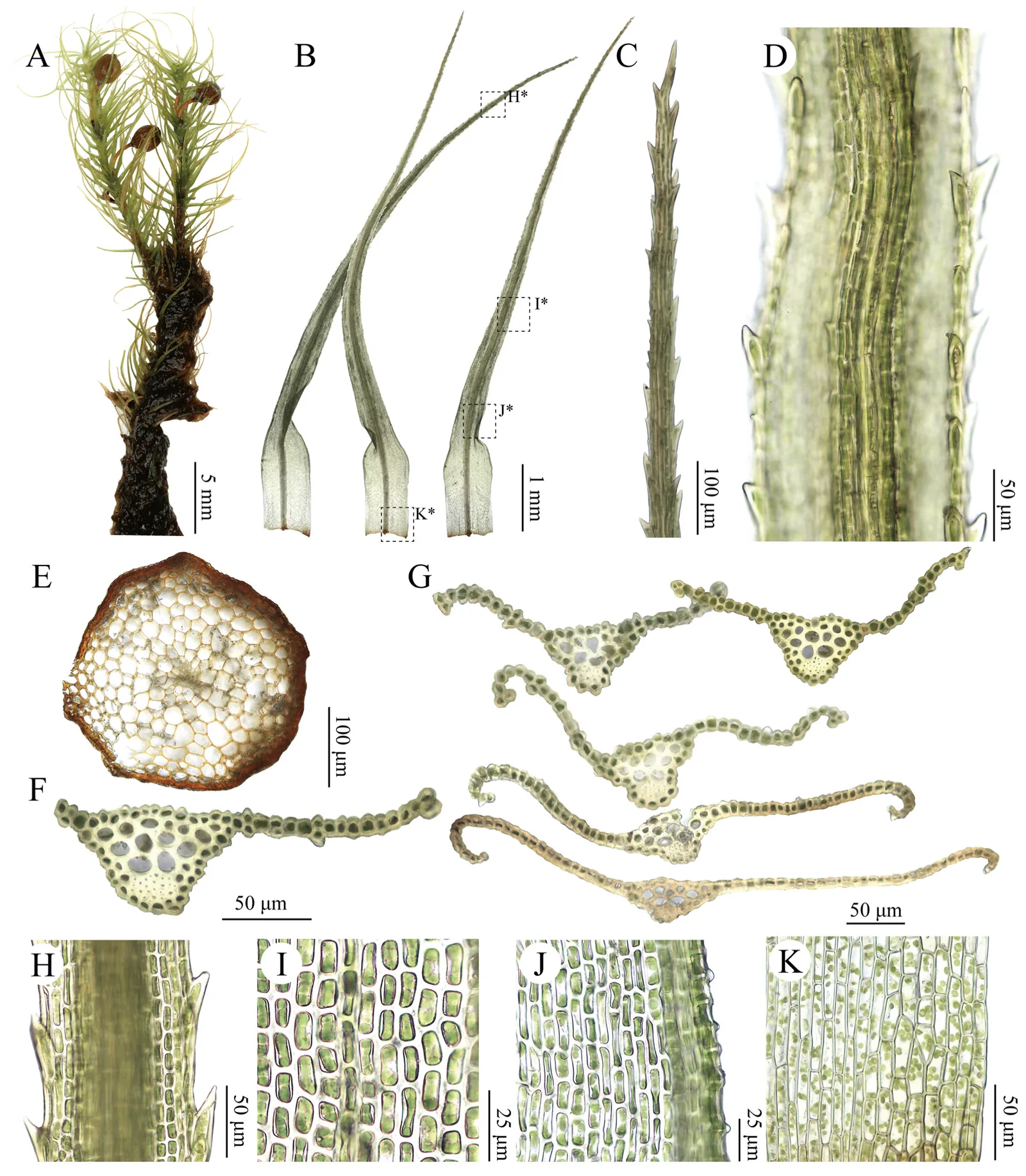
*Bartramia halleriana* Hedw. (**A**) Moist plants with mature sporophytes. (**B**) Leaves. (**C**) Leaf apex. (**D**) Abaxial costal cells at mid-leaf. (**E**) Transverse section of stem. (**F**) Transverse section of the mid-leaf. (**G**) Transverse sections of leaves. (**H**) Upper laminal cells. (**I**) Median laminal cells. (**J**) Basal laminal cells. (**K**) Alar cells. (**H**), (**I**), (**J**), and (**K**) are enlarged details of H*, I*, J*, and K*, respectively. All from *W.-Z. Huang 20250825-01* (HTC).

**Figure 6:**
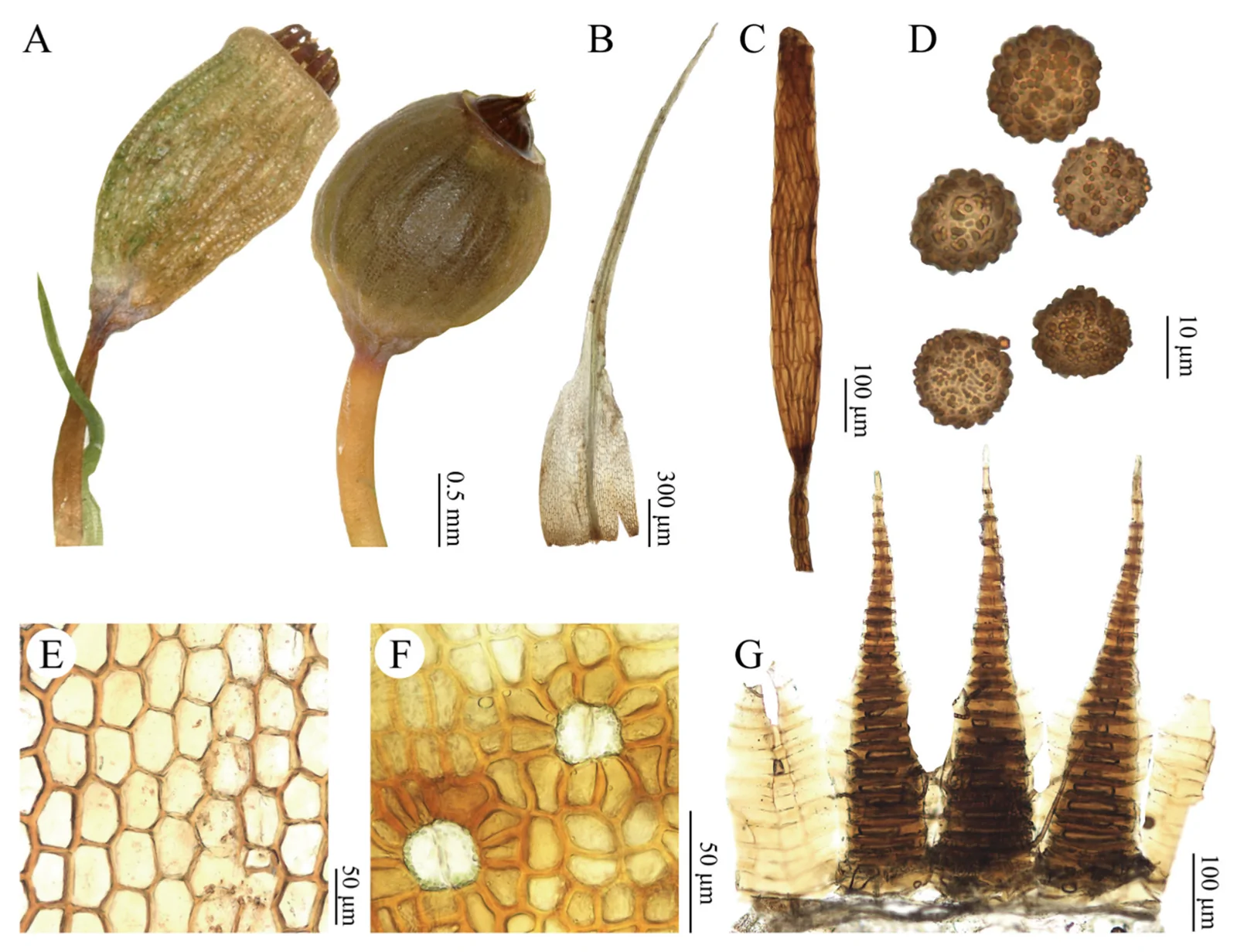
*Bartramia halleriana* Hedw. (**A**) Capsules, dry and moist. (**B**) Perichaetial leaves. (**C**) Antheridia. (**D**) Spores. (**E**) Exothecial cells. (**F**) Stomata. (**G**) Peristome. All from *W.-Z. Huang* 20250825-01 (HTC).

Notes: *Bartramia halleriana* is morphologically similar to *B. pomiformis* in having leaves that are indistinctly differentiated into a distinct sheathing base and limb, revolute leaf margins, and a double peristome [3] (Figure 5B–G, Figure 6G, Figure 10B–G and Figure 11G). However, the two species can be distinguished by the following characters: (1) in *B. halleriana*, the setae are short, usually no more than twice as long as the capsule length and generally less than 5 mm long; consequently, the capsules are often concealed among the leaves [3,6,27,30,37,38] (Figure 5A and Figure 6A). In contrast, the setae of *B. pomiformis* are several times as long as the capsules and generally more than 5 mm long; the capsules are therefore clearly exserted above the leaves [3,6,27,30,37] (Figure 10A and Figure 11A); (2) the leaves of *B. halleriana* are usually setaceous distally and are not strongly flexuose or curled when dry [3,6,27,37,38] (Figure 5A–C), whereas those of *B. pomiformis* are more slenderly lanceolate and usually flexuose to strongly curled when dry [3,6,27,30,37] (Figure 10A–C).

#### 2.1.4. *Bartramia ithyphylla* Brid., Muscol. Recent. 2(3): 132. 1803. (Figure 7 and Figure 8)

Chinese name: 直叶珠藓 (Zhí Yè Zhū Xiǎn)

Type: SWITZERLAND. “Helvetia” (lectotype: B, designated by Fransén [2], not seen).

**Figure 8:**
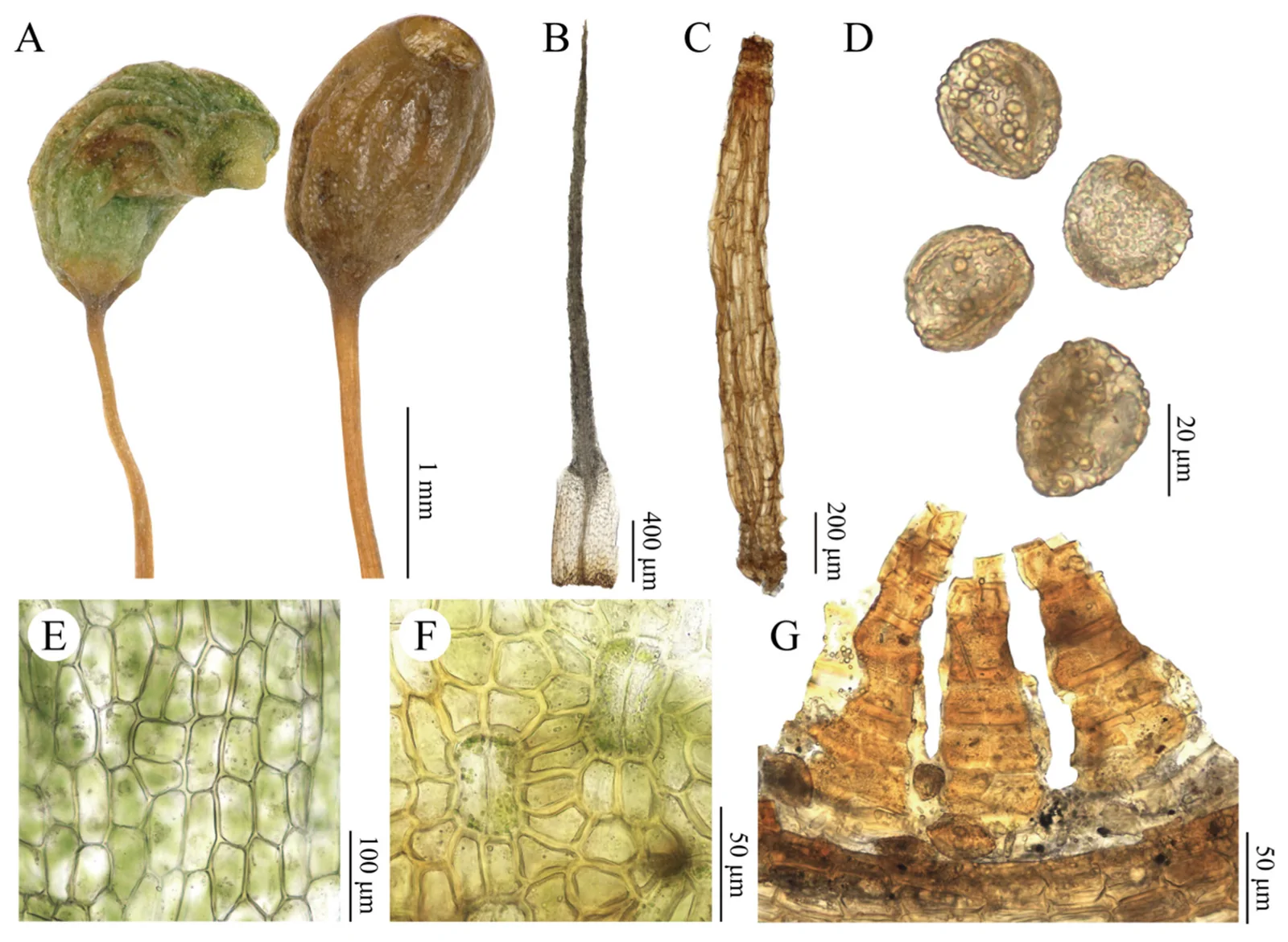
*Bartramia ithyphylla* Brid. (**A**) Capsules, dry (**left**) and moist (**right**). (**B**) Perichaetial leaves. (**C**) Antheridia. (**D**) Spores. (**E**) Exothecial cells. (**F**) Stomata. (**G**) Peristome. All from *W.-Z. Huang et al. 2025072736* (HTC).

Description: Plants excluding sporophytes 1.2–3 cm tall, forming dense tufts, with few to dense reddish-brown rhizoids. Stems 0.2–0.3 mm in diameter, with a differentiated central strand occupying 13–15% of the stem diameter. Leaves 3.5–4.2 mm long, erect-appressed when dry, spreading when moist, differentiated into a hyaline sheathing base and a subulate limb. Sheathing base 0.8–0.9 mm long, 0.4–0.7 mm wide at shoulders, hyaline, unistratose; cells smooth, hyaline, elongate-rectangular to linear, 50.5–155.5 × 4.0–17.5 μm. Costa in the sheathing base distinct; in transverse section with one layer of large guide cells, weakly developed stereid bands, and differentiated dorsal and ventral epidermal cells. Limb 2.6–3.3 mm long, 0.3–0.4 mm wide at base; limb cells linear to narrowly rectangular, 16.5–57.5 × 1.5–5.0 μm, with low mammillae. Costa in the limb diffuse, without spines; in transverse section with one layer of large guide cells, stereid group small, and differentiated dorsal and ventral epidermal cells. Margins plane, serrulate.

Synoicous. Antheridia clavate, borne among archegonia; archegonia flask-shaped, with a slender neck and swollen venter. Perichaetial leaves shorter than or as long as vegetative leaves. Setae 6.4–7.0 mm long. Capsules inclined, asymmetric, 1.8–1.9 mm long, 0.9–1.1 mm in diameter; exothecial cells irregularly rectangular, (39.0–)66.5–104.5(–126.0) μm long and (20.5–)24.5–46.5(–59.0) μm wide, thin-walled. Stomata restricted to the capsule base, superficial. Peristome double; exostome teeth yellowish-brown, fragmentary in observed capsules, transversely trabeculate, finely papillose; endostome with a basal membrane present, segments fragmentary but evident, finely papillose to weakly striolate-papillose; cilia not observed. Spores (15.0–)20.5–31.5 μm in diameter, globose to subglobose, yellowish-brown to brown, coarsely verrucose.

Distribution. China: Anhui, Chongqing, Fujian, Gansu [33], Guangdong [32], Guangxi, Guizhou, Heilongjiang, Henan, Hubei [34], Inner Mongolia, Jilin, Liaoning, Shaanxi, Sichuan, Taiwan [35], Xinjiang, Xizang (new record), Yunnan [11], and Zhejiang [15,22,23,24].

Notes: *Bartramia ithyphylla* is morphologically most similar to *B. deciduifolia*, particularly in having plane limb margins and a small, diffuse group of costal stereids at mid-limb. The two species can be distinguished by the following characters: (1) the leaves remain attached in *B. ithyphylla* [2,6,27,37,38] (Figure 7A), whereas leaves are usually deciduous in *B. deciduifolia* [2,37] (Figure 3A); (2) the guide cells of the costa are arranged in a single layer in *B. ithyphylla* (Figure 7F,G), but in two or three layers in *B. deciduifolia* (Figure 3F,G); and (3) the costa is indistinct within the limb in *B. ithyphylla* [2,27,37] (Figure 7B,F–H), but more or less prominent in *B. deciduifolia* [2] (Figure 3B,F–H).

#### 2.1.5. *Bartramia laevisphaera* (Taylor) Müll. Hal., Syn. Musc. Frond. 1: 506. 1849. (Figure 9)

Chinese name: 平果珠藓 (Píng Guǒ Zhū Xiǎn)

Type: ECUADOR. Pichincha, on shaded tree trunks and on the ground, 23 September 1826, *W. Jameson 1827* (lectotype: FH, isolectotype: LIL-Matteri. Designated by Suárez et al. [39]; not seen).

Description: Plants excluding sporophytes 1.1–3.6 cm tall, with few to dense reddish-brown rhizoids. Stems ca. 0.3 mm in diameter, erect or ascending, monopodially or dichotomously branched, with a central strand occupying ca. 20% of the stem diameter, lower portions with few to dense reddish-brown rhizoids. Leaves 4.2–5.1 mm long, erect-appressed when dry, erect-patent to spreading when moist, ovate-lanceolate to lanceolate, gradually narrowed from base to apex, not differentiated into a sheathing base and limb. Leaf apex gradually subulate, with the costa excurrent into a short to long spinulose awn. Leaf base concave, green, 0.5–0.7 mm wide; lamina mostly bistratose in the distal part, sometimes 2–3-stratose near the costa, becoming unistratose towards the base. Costa distinct, toothed abaxially; in transverse section with 1–2 layers of guide cells and a strong dorsal stereid band. Margins revolute, coarsely double-serrate above. Upper laminal cells short-rectangular to linear, 7.5–35.0 × 2.5–10.0 μm, papillose; median cells rectangular to linear, 9.0–41.5 × 3.5–13.0 μm; basal cells linear to elongate-rectangular, 12.5–100.5 × 3.0–10.5 μm.

Sporophytes not observed.

Distribution. China: Qinghai (new record), Sichuan [24], Taiwan [15,22,23,24,35], Xizang (new record).

**Figure 9:**
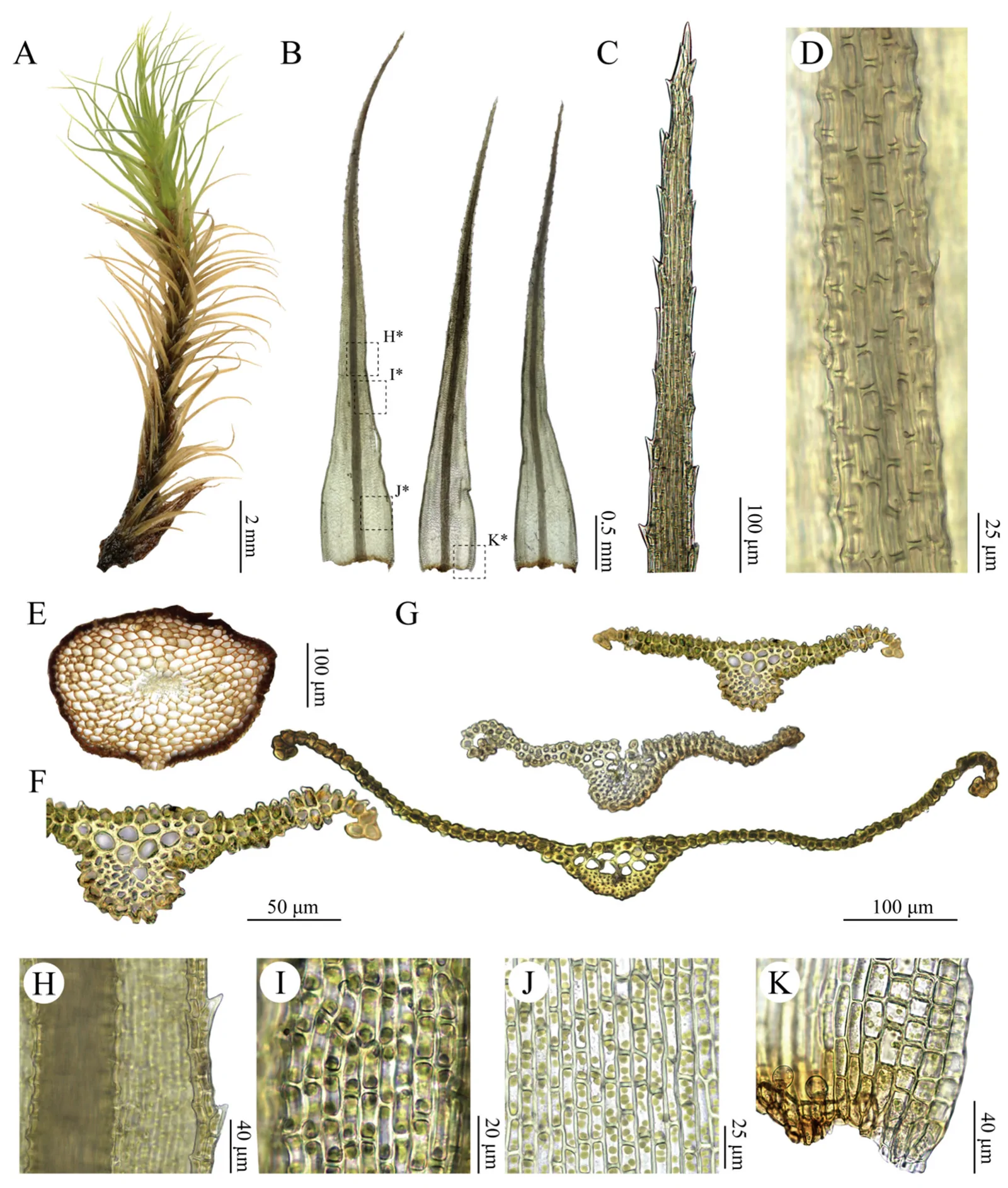
*Bartramia laevisphaera* (Taylor) Müll. Hal. (**A**) Moist plants. (**B**) Leaves. (**C**) Leaf apex. (**D**) Abaxial costal cells at mid-leaf. (**E**) Transverse section of stem. (**F**) Transverse section of the mid-leaf. (**G**) Transverse sections of leaves. (**H**) Upper laminal cells. (**I**) Median laminal cells. (**J**) Basal laminal cells. (**K**) Alar cells. (**H**), (**I**), (**J**), and (**K**) are enlarged details of H*, I*, J*, and K*, respectively. All from *W.-Z. Huang 20250825-18* (HTC).

Notes: *Bartramia laevisphaera* can be distinguished from the other Chinese *Bartramia* species by the following combination of characters: (1) the upper lamina is predominantly bistratose and sometimes 2–3-stratose near the costa [6,27,40] (Figure 9F,G); (2) the leaves are not differentiated into a hyaline sheathing base and green subulate limb [5,6,27,40] (Figure 9B); and (3) the setae are short and bear subglobose, rugulose, gymnostomous capsules [27,40].

*Bartramia laevisphaera* was originally described as *Glyphocarpa laevisphaera* by Taylor [41] and was subsequently transferred to *Bartramia* by Müller [42]. Flowers [40,43] later treated the species as *Anacolia laevisphaera*, mainly on the basis of its strong costa, partly bi- to multistratose lamina, short seta, subglobose gymnostomous capsule, and coarsely papillose spores. This placement was adopted in subsequent floristic treatments, including the checklist of Chinese mosses, in which the species was reported from Taiwan as *Anacolia laevisphaera* [15]. Molecular phylogenetic analyses subsequently recovered *A. laevisphaera* within the *Bartramia* sect. *Strictidium* lineage, supporting its placement in *Bartramia* [13].

In China, Iwatsuki and Sharp [12] listed one specimen of *Bartramia laevisphaera* from Taiwan. Subsequently, Li et al. [6] listed two additional specimens from Sichuan Province. We additionally found two specimens of this species from Qinghai and Xizang Province. Therefore, this species has been known from five localities since it was first recorded in China 57 years ago. Present data estimate the area of occupancy (AOO) of *B. laevisphaera* to be 20 km^2^, which falls within the limits of the critically endangered (EN) category under the subcriterion B2 (AOO less than 500 km^2^ and known to exist at no more than five locations). Notably, this species has so far been found only at high elevations in China. High-altitude habitats suitable for rare, endemic, or threatened species are more vulnerable to climate change and human-induced landscape alteration [44,45,46]. Therefore, we propose to assign this species to the endangered (EN) category due to its small population and habitat (criteria B2ab(ii, iii)), based on [47,48,49].

#### 2.1.6. *Bartramia pomiformis* Hedw., Sp. Musc. Frond.: 164. 1801. (Figure 10 and Figure 11)

Chinese name: 梨蒴珠藓 (Lí Shuò Zhū Xiǎn)

Type: Europe according to protologue; locality not indicated on the specimen; first specimen from the left in the upper row on the Hedwig–Schwägrichen sheet (lectotype: G [G00040043], designated by Ederra [31], not seen).

Description: Plants excluding sporophytes 1.8–3.0 cm tall, with well-developed reddish-brown rhizoids. Stems 0.3–0.4 mm in diameter, simple or sparsely branched, with a central strand occupying ca. 16–17% of stem diameter, lower portions with well-developed reddish-brown rhizoids. Leaves 5.0–8.3 mm long, usually flexuose to strongly curled when dry, patent to spreading when moist, narrowly lanceolate to linear-lanceolate, gradually narrowed from base to apex, rather indistinctly differentiated into a sheathing base and limb. Leaf base erect, 0.4–0.6 mm wide; lamina unistratose. Costa percurrent to excurrent, prominent, smooth to weakly rough abaxially; in transverse section with two layers of guide cells and a well-developed stereid group. Margins revolute, serrulate to double-serrate distally. Upper laminal cells quadrate to short-rectangular, 2.0–18.5 × 3.5–10 μm, papillose; median cells short-rectangular to rectangular, 6.0–18.5 × 2.5–8.0 μm; basal cells linear to elongate-rectangular, 11–50 × 2–10 μm.

Synoicous. Antheridia clavate; archegonia flask-shaped; Perichaetial leaves shorter than vegetative leaves, triangular to ovate-lanceolate. Setae 6.0–15.0 mm long, straight. Capsules inclined, irregular, globose to slightly oblong, 1.9–2.1 mm long, 1.3–1.7 mm in diameter, furrowed when dry; exothecial cells irregularly hexagonal, rectangular to quadrate, 33.0–89.5 μm long and 20.5–68.0 μm wide, thick-walled. Stomata restricted to the capsule base, phaneropore. Peristome double; exostome teeth yellowish-brown, transversely trabeculate, finely to coarsely papillose; endostome well developed, with a basal membrane and finely papillose to striolate-papillose segments; cilia not observed. Spores 15.5–25.0 μm in diameter, spherical to subglobose, dark brown, coarsely papillose.

Distribution. China: Anhui, Chongqing, Fujian, Gansu [33], Guangdong [32], Guizhou, Hebei, Heilongjiang, Henan, Hubei [34], Hunan, Inner Mongolia [50], Jiangsu, Jiangxi, Jilin, Liaoning, Qinghai (new record), Shaanxi, Sichuan, Taiwan [35], Xinjiang, Yunnan [11], and Zhejiang [15,22,23,24].

Notes: *Bartramia pomiformis* is morphologically similar to *B. halleriana*; detailed diagnostic differences between the two species are provided in the notes under *B. halleriana*.

**Figure 10:**
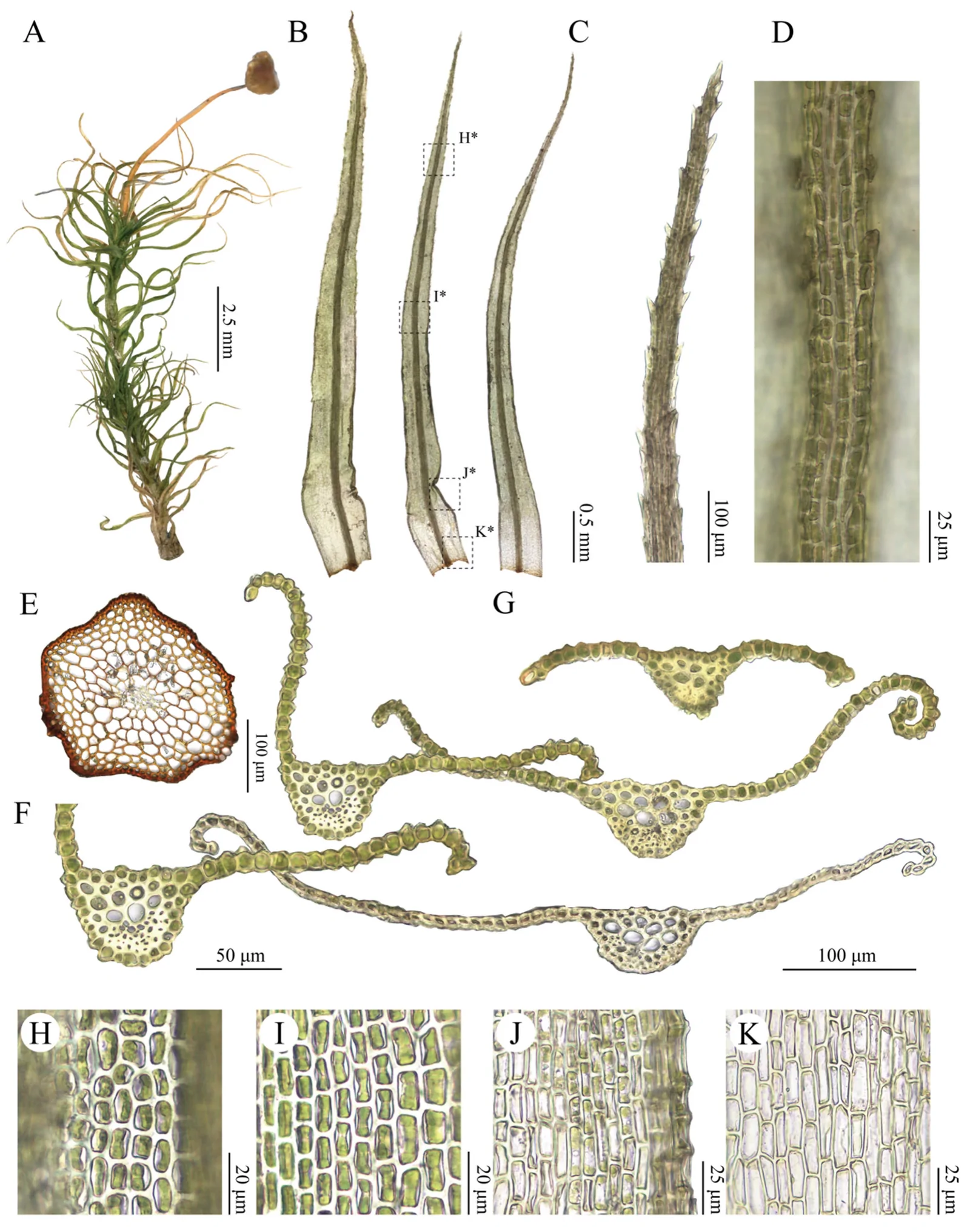
*Bartramia pomiformis* Hedw. (**A**) Moist plants with mature sporophytes. (**B**) Leaves. (**C**) Leaf apex. (**D**) Abaxial costal cells at mid-leaf. (**E**) Transverse section of stem. (**F**) Transverse section of the mid-leaf. (**G**) Transverse sections of leaves. (**H**) Upper laminal cells. (**I**) Median laminal cells. (**J**) Basal laminal cells. (**K**) Alar cells. (**H**), (**I**), (**J**), and (**K**) are enlarged details of H*, I*, J*, and K*, respectively. All from *W.-Z. Ma 15977* (KUN).

**Figure 11:**
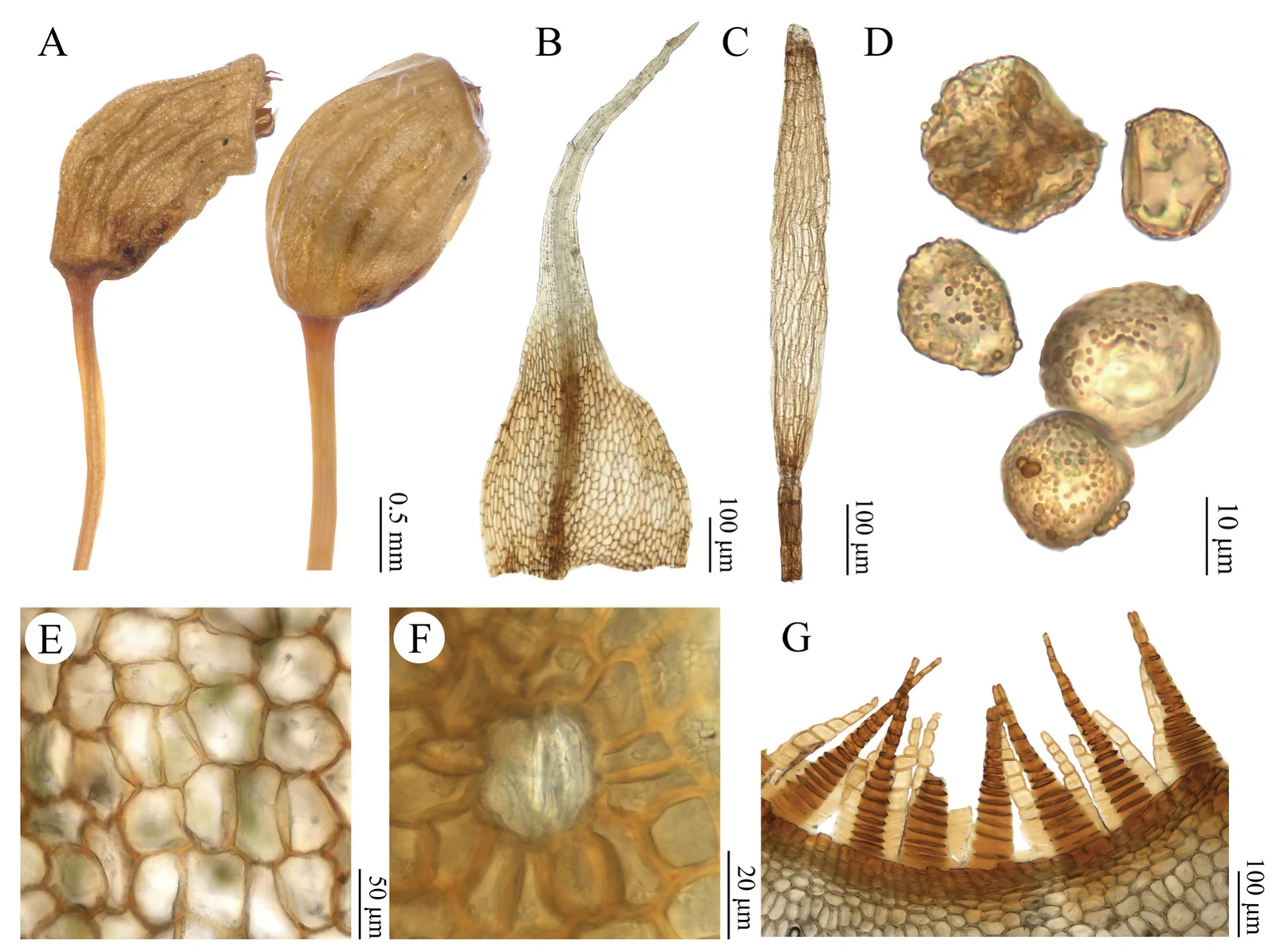
*Bartramia pomiformis* Hedw. (**A**) Capsules, dry and moist. (**B**) Perichaetial leaves. (**C**) Antheridia. (**D**) Spores. (**E**) Exothecial cells. (**F**) Stomata. (**G**) Peristome. All from *W.-Z. Ma 15977* (KUN).

#### 2.1.7. *Bartramia subulata* Bruch & Schimp., Bryol. Eur. 4: 53. 1846. (Figure 12 and Figure 13)

Chinese name: 绿珠藓 (Lǜ Zhū Xiǎn)

Type: AUSTRIA. Salzburg, Pinzgau, summit of Mt Gaisstein, “In summo cacumine montis Gaisstein Pinzgaviae, alt. 8000′” [ca. 2440 m], summer 1843, *W.P. Schimper s.n.* (holotype: BM, not seen).

Description: Plants small, excluding sporophytes, 0.2–1.1 cm tall, with few to dense reddish-brown rhizoids. Stems simple, 0.2–0.4 mm in diameter, with a differentiated central strand occupying ca. 30–50% of stem diameter. Leaves 2.9–3.4 mm long, erect-appressed when dry, erect to slightly spreading when moist, differentiated into a hyaline sheathing base and a green subulate limb. Sheathing base 0.7–0.9 mm long, 0.4–0.6 mm wide at shoulders, hyaline, unistratose; cells smooth, hyaline, linear to elongate-rectangular, 44–153.5 μm long and 8–25 μm wide. Limb subulate, 1.9–2.4 mm long, with marginal border absent or unistratose and up to 3 cell rows wide; limb cells linear to narrowly rectangular, 18–50 μm long and 2.5–8 μm wide, with high mammillae. Costa in the limb diffuse, rough abaxially; in transverse section with one layer of large guide cells and a small, weakly differentiated stereid group. Margins plane, serrulate.

Synoicous. Antheridia clavate, borne among archegonia; archegonia flask-shaped, with a slender neck and swollen venter. Perichaetial leaves ovate-lanceolate to lanceolate, shorter than to equalling vegetative leaves, gradually narrowed to an acuminate apex. Setae 5–9 mm long. Capsules erect, symmetric, ovoid, 1.7–2.1 mm long, 0.9–1.3 mm in diameter, furrowed when dry; exothecial cells irregularly rectangular to polygonal, 15–59 μm long and 19–32 μm wide, thin-walled. Stomata restricted to the capsule base, superficial. Peristome lacking. Spores 26–38 μm in diameter, ochraceous to brown, densely and finely papillose.

Distribution. China: Guizhou [24], Qinghai (new record), Shaanxi [15,22], Xizang, and Yunnan [11,22,23,24].

**Figure 12:**
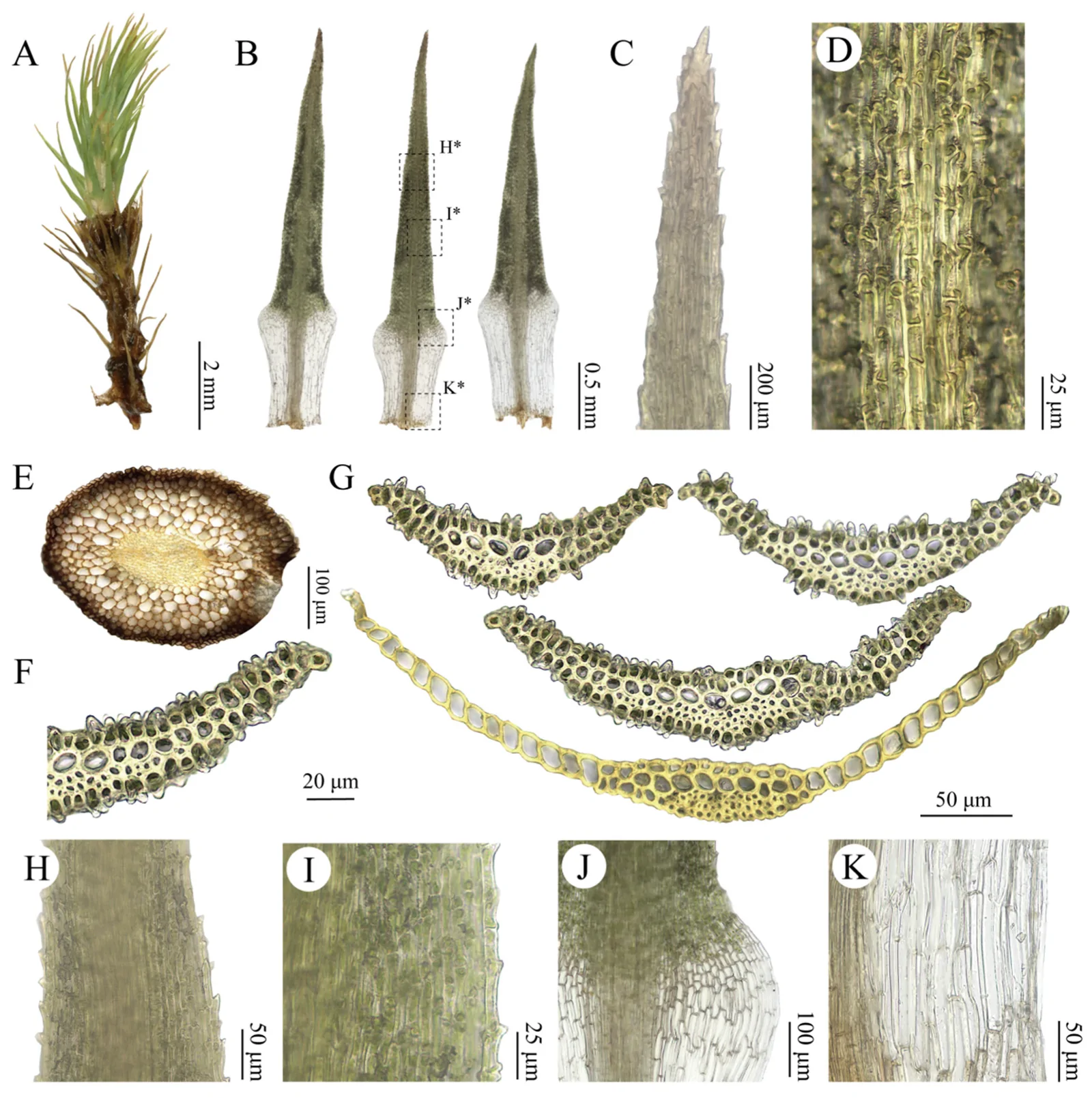
*Bartramia subulata* Bruch & Schimp. (**A**) Moist plants. (**B**) Leaves. (**C**) Leaf apex. (**D**) Abaxial costal cells of the limb. (**E**) Transverse section of stem. (**F**) Transverse section of the mid-limb. (**G**) Transverse sections of leaves. (**H**) Upper limb cells. (**I**) Basal limb cells. (**J**) Shoulder cells. (**K**) Sheathing-base cells. (**H**), (**I**), (**J**), and (**K**) are enlarged details of H*, I*, J*, and K*, respectively. All from *W.-Z. Huang 20250823-7* (HTC).

**Figure 13:**
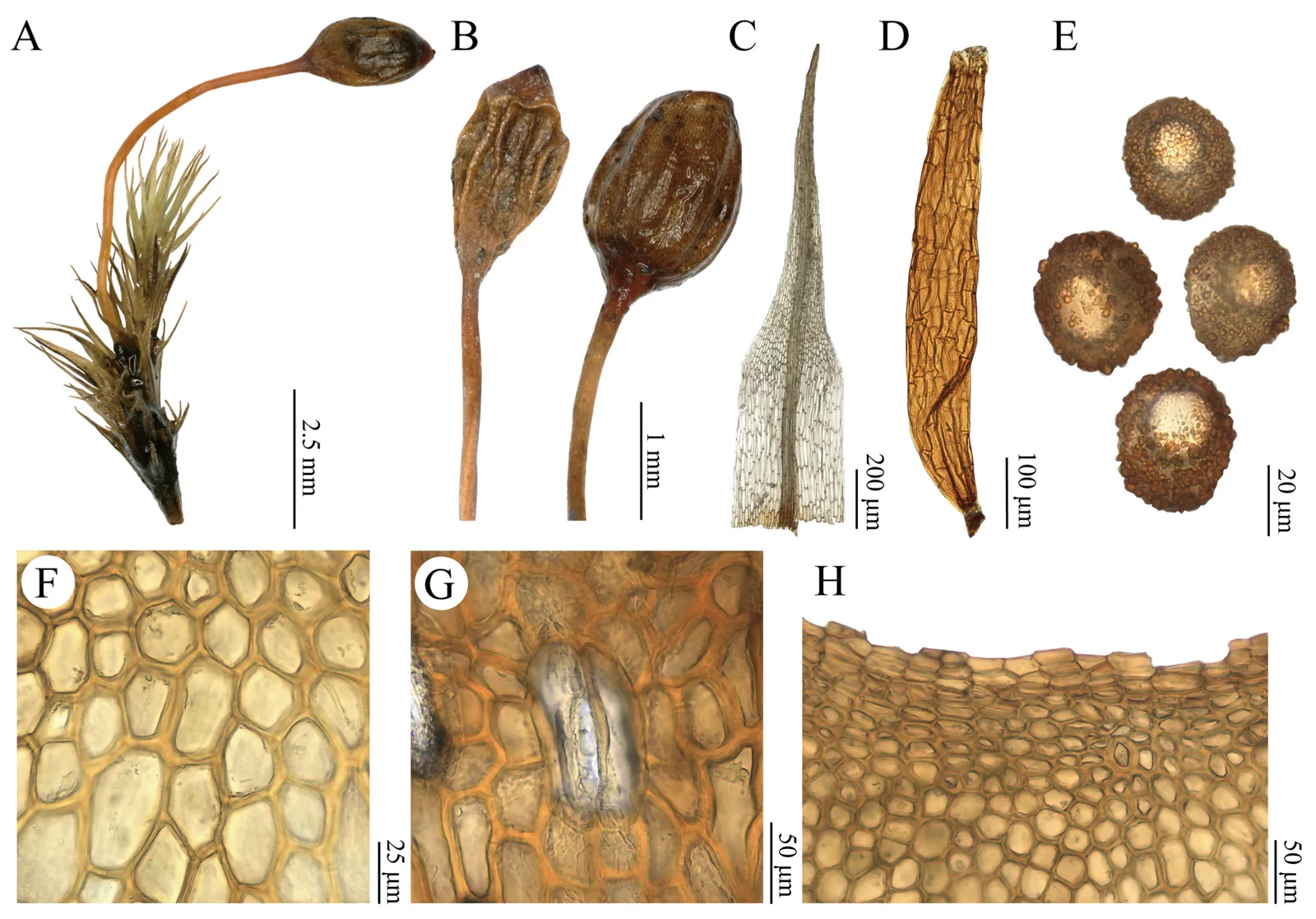
*Bartramia subulata* Bruch & Schimp. (**A**) Plants with mature capsules. (**B**) Capsules, dry and moist. (**C**) Perichaetial leaves. (**D**) Antheridia. (**E**) Spores. (**F**) Exothecial cells. (**G**) Stomata. (**H**) Capsule mouth showing absence of peristome. All from *W.-Z. Huang 20250823-7* (HTC).

Notes: *Bartramia subulata* is readily distinguished from the other Chinese species of *Bartramia* by the following combination of characters: short, subulate leaves that are usually less than 3.5 mm long and erect-appressed when dry [2,27] (Figure 12A–C); small, erect, symmetric capsules [2,6,27] (Figure 13A,B); and the absence of a peristome [2,27] (Figure 13H). The species occurs primarily in alpine habitats at or above timberline [2,27].

### 2.2. Excluded Species

*Bartramia potosica* Mont., Ann. Sci. Nat., Bot., sér. 2, 9(1): 56. 1838.

Reported as *Bartramia fragilifolia* in China by Li [14], but Redfearn et al. [15] noted that this species, distributed in America, was doubtful. It was subsequently not included in the Moss Flora of China by Li et al. [6]. We examined all specimens collected by Li and deposited in KUN, but none could be referred to *B. fragilifolia*. Therefore, the species should be excluded from the bryoflora of China.

### 2.3. Key to the Chinese Species of Bartramia

1. Leaves not or indistinctly differentiated into a hyaline sheathing base and a green subulate limb; shoulders absent or weakly differentiated............................................................2
1. Leaves distinctly differentiated into a hyaline sheathing base and a green subulate limb; shoulders distinct ...........................................................................................................................4
2. Upper lamina bistratose, sometimes 2–3–stratose near the costa...................*B. laevisphaera*
2. Upper lamina unistratose or only partly bistratose...............................................................3
3. Setae short, usually no more than twice as long as the capsules and generally less than 5 mm long; the capsules are usually concealed among the leaves; leaves flexuose, not strongly curled when dry............................................................................................*B. halleriana*
3. Setae elongate, usually several times as long as the capsules and generally more than 5 mm long; the capsules are clearly exserted above the leaves; leaves usually strongly curled when dry.....................................................................................................................*B. pomiformis*
4. Limb/sheath ratio < 2.5, leaves usually less than 3.5 mm long; capsules gymnostomous.................................................................................................................................*B. subulata*
4. Limb/sheath ratio > 2.5, leaves usually longer than 3.5 mm, capsules with a single or double peristome............................................................................................................................5
5. Cross section of the costa with one layer of guide cells........................................*B. ithyphylla*
5. Cross section of the costa with two or three layers of guide cells..........................................6
6. Leaves usually deciduous; limb cells with low mammillae; limb margins usually plane or slightly recurved....................................................................................................*B. deciduifolia*
6. Leaves not deciduous; limb cells with high and distinct mammillae; limb margins more or less revolute...............................................................................................................*B. brevifolia*

## 3. Materials and Methods

In total, 120 specimens from 12 Chinese provinces were examined (Appendix A) including 73 specimens collected by the authors and deposited in the Herbarium of Hangzhou Normal University (HTC), and 47 earlier collections deposited in the following Chinese herbaria: Guizhou Normal University (GNUB), Huanggang Teachers College (HGTC), East China Normal University (HSNU), Institute of Applied Ecology, Chinese Academy of Sciences (IFP), Kunming Institute of Botany, Chinese Academy of Sciences (KUN), Lushan Botanical Garden, Chinese Academy of Sciences (LBG), Institute of Botany, Chinese Academy of Sciences (PE), Fairy Lake Botanical Garden, Shenzhen & Chinese Academy of Sciences (SZG), Xinjiang University (XJU), and Tibet Plateau Institute of Biology (XZ). Plants were examined using a Leica stereo microscope (Leica EZ4; Leica, Wetzlar, Germany) and an Olympus compound microscope (Olympus BX51; Olympus, Tokyo, Japan). Macroscopic images were captured with a digital microscope (Keyence VHX-6000, Tokyo, Japan), and microscopic images were captured with a MOTICAM S6 digital camera mounted on the compound microscope. All morphological descriptions in this study were based on the Chinese specimens examined.

The distribution of *Bartramia* species in China was compiled using literature, herbaria, open-access databases (e.g., National Plant Specimen Resource Center, https://www.cvh.ac.cn/), and our field data in recent years. The extent of occurrence (EOO) and area of occupancy (AOO) were calculated using GeoCAT [51], and the risk of extinction of the species was assessed using the International Union for Conservation of Nature (IUCN) Red List Categories and Criteria [47,48,49].

## 4. Conclusions

This study elucidates the species diversity, geographic distribution, and conservation status of *Bartramia* in China through an examination of recent field collections and herbarium specimens. Seven species are recognized, including *B. deciduifolia*, which is newly recorded for China, with confirmed occurrences in Qinghai, Sichuan, Xizang, and Yunnan. Reassessment of the available specimens also provides a more robust basis for delineating the distribution of the genus within China. Of seven recognized species, *B. laevisphaera* is assessed as Endangered [EN B2ab(ii,iii)], whereas the remaining six are categorized as Least Concern. Collectively, the revised morphological descriptions, illustrations, distributional information, conservation assessments, and identification key establish an updated taxonomic framework for the identification and future study of *Bartramia* in China and provide a scientific foundation for future conservation assessments and planning.

## Figures and Tables

**Figure 4:**
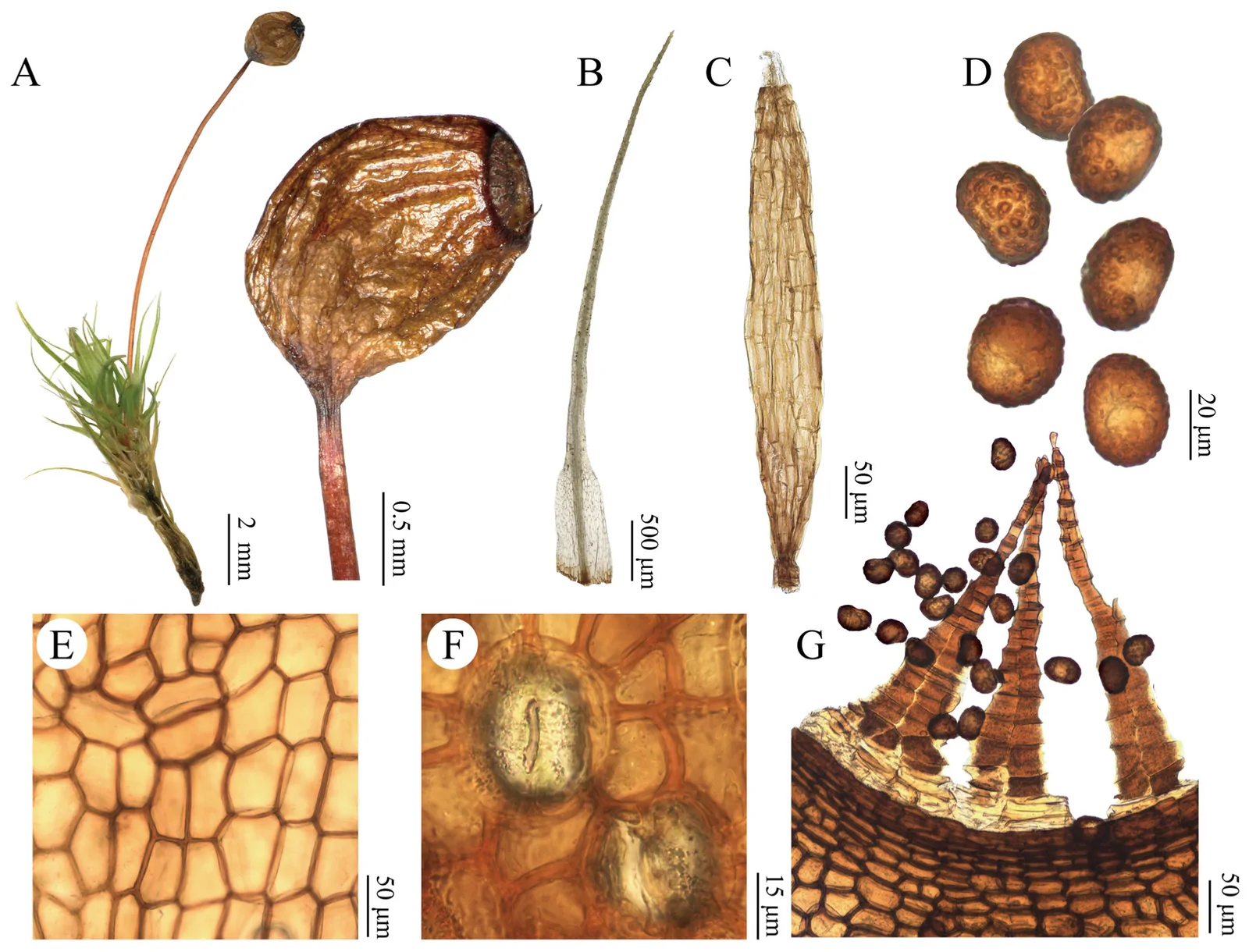
*Bartramia deciduifolia* Broth. & Yasuda. (**A**) Plants with mature capsules. (**B**) Perichaetial leaves. (**C**) Antheridia. (**D**) Spores. (**E**) Exothecial cells. (**F**) Stomata. (**G**) Peristome. All from *W.-Z. Huang 2025092806* (HTC).

## Data Availability

The data presented in this study are available within the article. Voucher specimens examined in this study are deposited in the herbaria indicated in the Materials and Methods and Specimens examined sections.

## Appendix A. Specimens Studied

***Bartramia brevifolia***

**CHINA. Yunnan:** Dali Bai Autonomous Prefecture, Dali City, Cangshan Mountain, Zhonghe Temple, on rock, 2580 m a.s.l., 30 August 2005, *L.-S. Wang et al. 05-25162* (KUN).

***Bartramia deciduifolia***

**CHINA. Qinghai:** Guoluo Zangzu Autonomous Prefecture, Jiuzhi County, Nianbaoyuze Nature Reserve, beside a grassland stream, on soil, 33°21′57.96″ N, 101°04′16.21″ E, 4295 m, 28 August 2020, *C. Shen et al. 20200828-9B* (HSNU). **Sichuan:** Aba Tibetan and Qiang Autonomous Prefecture, Xiaojin County, Siguniangshan Town, Siguniangshan Scenic Area, Changpinggou Valley, from Muluozi Campsite to Sigula Temple, on soil, 31°06′23.22″ N, 102°52′02.35″ E, 3615 m, 25 June 2026, *W.-Z. Huang 20260625-19* (HTC). **Xizang:** Linzhi City: Bayi District, Cuomujiri Scenic Area, on soil, 29°48′35.95″ N, 94°25′20.31″ E, 4128 m, 30 September 2025, *W.-Z. Huang 2025093012* (HTC); Bayi District, Lulang Town, National Highway G318, on soil, 29°38′32.23″ N, 94°41′51.28″ E, 4003 m, 28 September 2025, *W.-Z. Huang 2025092806* (HTC); Bayi District, Mirui Township, Linda Line, on soil, 29°27′14.00″ N, 94°31′13.00″ E, 4000 m, 25 July 2020, *H.-P. Ma 2020072591G1016* (XZ); Bayi District, forest near National Highway G318, on soil, 29°38′27.70″ N, 94°41′51.96″ E, 4026 m, 23 September 2020, *Y.-L. Xiang et al. 20200923-78B* (HSNU); Motuo County, Bangxing Township, wetland below 55K Qinshuidie Waterfall, on soil, 29°42′58.39″ N, 95°36′53.78″ E, 2994 m, 27 September 2025, *W.-Z. Huang 20250927224* (HTC); Motuo County, Dexing Township, Paimo Road, at the exit of Xiongdola Tunnel, on rock and soil, 29°28′42.47″ N, 94°58′12.42″ E, 3575 m, 10 October 2024, *W.-Z. Huang & F.-Y. Zhang 20241010-6* (HTC); ibid., *W.-Z. Huang & F.-Y. Zhang 20241010-9* (HTC); Motuo County, Dexing Township, Xiaoyandong, along road trail, on rock, 29°24′56.52″ N, 95°04′07.88″ E, 2691 m, 12 October 2024, *W.-Z. Huang & F.-Y. Zhang 20241012-78* (HTC); Motuo County, Dexing Township, Xiaoyandong, along the gorge down to the riverside, on rock, 29°24′48.70″ N, 95°04′09.95″ E, 2640 m, 12 October 2024, *W.-Z. Huang & F.-Y. Zhang 20241012-32* (HTC); Motuo County, vicinity of the 52 km milestone from Bomi to Motuo, on rock in sparse forest, 29°44′22.0″ N, 95°40′43.1″ E, 3310 m, 22 June 2014, *L. Zhang & Q. Zuo 11866* (SZG); Rikaze City: Nielamu County, Zhangmu Town, midway from Lixin to Xuebugang, on rock in forest, 27°56′59.8″ N, 85°58′44.3″ E, 2750 m, 5 August 2012, *L. Zhang 9259* (SZG 00066262); ibid., on soil, *L. Zhang 9266* (SZG 00066270); Yadong County, Xiasima Town, high-elevation hillside, on soil, 27°36′46.20″ N, 88°50′15.97″ E, 4395 m, 20 September 2020, *Y.-L. Xiang et al. 20200920-55* (HSNU). **Yunnan:** Dali Bai Autonomous Prefecture, Dali City, Cangshan Mountain, along the road to the television relay station, 25°41′32″ N, 100°07′00″ E, 3589 m, 8 August 2010, *W.-Z. Ma & Z.-J. Yin 10-1715* (KUN); Dali Bai Autonomous Prefecture, Heqing County, Ma’er Mountain, near Yanchi, on soil, 26°15′31.31″ N, 100°06′28.04″ E, 3499 m, 29 May 2021, *W.-Z. Huang et al. 20210529-27B* (HSNU); Diqing Tibetan Autonomous Prefecture, Weixi Lisu Autonomous County, Yongchun Township, Tuozhi Village, on soil slope, 27°03′01″ N, 99°21′00″ E, 2886 m, 25 July 2010, *W.-Z. Ma & H.-J. Dong 10-1407* (KUN); Kunming City, Luquan Yi and Miao Autonomous County, Zhuanlong Town, Jiaozi Snow Mountain, under Rhododendron and Abies, 26°04′00.00″ N, 102°50′00.00″ E, 3750 m, 26 September 2006, *L.-S. Wang 06-27019* (KUN B0011498).

***Bartramia halleriana***

**CHINA. Hubei:** on rock on a hillside, 1800 m, 15 November 1980, *L.-J. Bi 30632* (IFP). **Qinghai:** Guoluo Zangzu Autonomous Prefecture, Banma County, Dengta Township, Bangcha Management Station, Bangcha Nursery, on soil, 32°39′28.94″ N, 101°00′02.22″ E, 3234 m., 25 August 2025, *W.-Z. Huang 20250825-23* (HTC); ibid., *W.-Z. Huang 20250825-29* (HTC); Banma County, Dengta Township, Gerize Gully, on soil, 32°44′22.47″ N, 101°10′29.30″ E, 3482 m, 26 August 2025, *W.-Z. Huang 20250826-15* (HTC); Banma County, Dengta Township, Gerize Gully, on soil, 32°40′56.85″ N, 101°08′00.35″ E, 3229 m, 26 August 2025, *W.-Z. Huang 20250826-7* (HTC); Banma County, Dengta Township, Mailangou, on soil, 32°45′52.34″ N, 100°55′34.63″ E, 3502 m, 25 August 2025, *W.-Z. Huang 20250825-01* (HTC); Banma County, Maheke Township, along X709 Road, on soil under shrubs, 33°05′03.60″ N, 100°25′21.14″ E, 3836 m, 23 August 2025, *W.-Z. Huang 20250823-3* (HTC); Banma County, on soil, *S.-B. Zhang 139* (HTC). **Sichuan:** Aba Tibetan and Qiang Autonomous Prefecture, Jiuzhaigou County, Jiuzhaigou National Nature Reserve, Zangmalongli Valley, in primary forest, on humus, 33°04′10.1″ N, 103°51′29.8″ E, 3030 m, 17 August 2011, *L. Zhang et al. 8204* (SZG); Aba Tibetan and Qiang Autonomous Prefecture, Xiaojin County, Siguniangshan Town, Siguniangshan Scenic Area, Changpinggou Valley, from Sigula Temple to Muluozi Campsite, on rock, 31°02′35.31″ N, 102°52′27.96″ E, 3420 m, 23 June 2026, *W.-Z. Huang 20260623-36* (HTC); ibid., on rock, 31°03′49.76″ N, 102°52′30.69″ E, 3527 m, 23 June 2026, *W.-Z. Huang 20260623-74* (HTC); Xiaojin County, Siguniangshan Town, Siguniangshan Scenic Area, Haizigou Valley, from the scenic-area ticket office to Chaoshanping, on soil, 30°59′52.20″ N, 102°50′39.20″ E, 3410 m, 27 June 2026, *W.-Z. Huang 20260627-14* (HTC); Ganzi Tibetan Autonomous Prefecture, Jiulong County, Tuozha Township, red birch forest, on thin soil over rock, 29°07′39.94″ N, 101°32′09.13″ E, 3463 m, 12 April 2021, *Q. He & Z.-Q. Yi 430* (PE); Kangding City, Gongga Mountain, on soil, 4 October 2024, *Y.-L. Xiang et al. 20241004-22* (GNUB); ibid., *Y.-L. Xiang et al. 20241004-34* (GNUB); Luding County, Hailuogou Glacier Forest Park, Hailuogou, Caohaizi, at the base of a tree beside a stone path, 29°35′11.06″ N, 102°01′36.08″ E, 2778 m, 17 April 2021, *Q.-H. Wang 2662* (PE); Leshan City, Emeishan City, Emei Mountain, Jinding, Right Heaven Gate, on limestone in sparse fir forest, 2900 m, 25 August 1978, *Q. Li 00002425* (IFP). **Xizang:** Linzhi City, Bayi District, Linzhi Town, Kangzha Village, National Highway G318, epiphytic on a tree, 29°33′45.00″ N, 94°34′31.00″ E, 3800 m, 26 July 2020, *H.-P. Ma 20200726P6h6014* (XZ); Linzhi City, Bayi District, Linzhi Town, Kangzha Village, National Highway G318, on soil, 29°33′41.14″ N, 94°34′36.45″ E, 3886 m, 23 September 2020, *Y.-L. Xiang et al. 20200923-14B* (HSNU); Linzhi City, Bayi District, Lulang Town, along Provincial Road S504 from Baga Village to Luomu Village, on soil, 30°00′26.14″ N, 94°40′24.05″ E, 2789 m, 29 September 2025, *W.-Z. Huang 20250929-120* (HTC); Linzhi City, Sejila Mountain, radar station, on soil, 29°37′01.00″ N, 94°39′55.00″ E, 4439.73 m, 18 June 2023, *H.-P. Ma 20230618-017* (XZ); Linzhi City, Bayi District, Lulang Town, National Highway G318, on soil, 29°38′32.23″ N, 94°41′51.28″ E, 4003 m, 28 September 2025, *W.-Z. Huang 20250928-08* (HTC); Linzhi City, Bomi County, Yigong Township, Chongsa Monastery, 10 km section on the Bomi–Motuo Road, on soil, 30°14′06.00″ N, 94°47′55.00″ E, 2450 m, 22 June 2023, *H.-P. Ma 20230622-7* (XZ); Linzhi City, Motuo County, Dexing Township, Xiaoyandong, Paimo Road, along the gorge down to the riverside, on rock, 29°24′55.37″ N, 95°04′15.61″ E, 2676 m, 14 October 2024, *W.-Z. Huang & F.-Y. Zhang 2024101454* (HTC); Motuo County, Mount Renqinbeng Zhuomala, pilgrimage circumambulation route, on soil, 29°18′15.41″ N, 95°20′32.68″ E, 2056 m, 18 October 2024, *W.-Z. Huang & F.-Y. Zhang 2024101838* (HTC); Motuo County, along Zhamo Highway (G219), on soil, 29°41′50.52″ N, 95°32′54.20″ E, 2702 m, 26 September 2025, *W.-Z. Huang 20250926-139* (HTC); Linzhi City, Zayu County, Cibagou National Nature Reserve, on rock in sparse forest, 28°39′04.33″ N, 97°03′19.15″ E, 2230 m, 17 August 2009, *L. Zhang 6570* (SZG 00076672); Linzhi City, Zayu County, Cibagou National Nature Reserve, on soil in forest, 28°39′04.33″ N, 97°03′19.15″ E, 2385 m, 17 August 2009, *L. Zhang 6601* (SZG 00076683); Linzhi City, Zayu County, Mingqi, along National Highway G219, on rock in forest, 28°48′09.36″ N, 97°36′31.45″ E, 3638 m, 13 July 2023, *H.-P. Ma 20230713-B-032* (XZ); Linzhi City, Zayu County, Nabang’le, on soil in forest, 28°53′28.76″ N, 97°44′52.30″ E, 3728 m, 24 July 2023, *H.-P. Ma 20230724-B-060* (XZ); Weila Township, on soil, 30°11′20.00″ N, 93°21′26.00″ E, 3708 m, *J.-Y. Mu 1593* (XZ). **Yunnan:** Lincang City, Yongde County, Yongde Daxueshan National Nature Reserve, ridge at Tieshanpo, on soil in a cave, 24°06′37″ N, 99°37′39″ E, 2830 m, *W.-Z. Ma 11-2659* (KUN); Nujiang Lisu Autonomous Prefecture, Gongshan Dulong and Nu Autonomous County, Dulongjiang Township, Dulongjiang National Park, on soil, 27°46′32.45″ N, 98°35′44.74″ E, 2236 m, *J.-L. Li GLG006* (HTC); Nujiang Lisu Autonomous Prefecture, Gongshan Dulong and Nu Autonomous County, ca. 6 km from the tunnel, along the road from Gongshan to Dulongjiang, toward an unnamed moraine lake, on SW-facing granite rock in a fissure, 27°45′14.00″ N, 98°27′18.00″ E, 3405 m, 12 September 2013, *W.-Z. Ma 13-4852* (KUN); Nujiang Lisu Autonomous Prefecture, Gongshan Dulong and Nu Autonomous County, Gaoligongshan region, along the Qiqi Trail on the eastern slope of Gaoligongshan, paralleling the Qiqi River between the first forestry buildings/village and the Qiqi River suspension bridge, mixed hardwood forest above the river, on a granitic rock wall in partial shade, 27°43′43.00″ N, 98°35′35.00″ E, 1870 m, *J.R. Shevock et al. 43208* (KUN 41533); Nujiang Lisu Autonomous Prefecture, Lanping Bai and Pumi Autonomous County, on the road up Xuebangshan, on a rock cliff, 26°28′14.00″ N, 99°30′50.00″ E, 3148 m, 30 July 2010, *W.-Z. Ma & Z.-H. Wang 10-1515* (KUN).

***Bartramia ithyphylla***

**CHINA. Xinjiang:** Altay Prefecture, Altay City, Hongdun Town, Xiaodonggou Forest Park, along Ahe Highway (G681), on soil, 48°00′16.96″ N, 88°18′20.53″ E, 1895 m, 19 July 2025, *W.-Z. Huang et al. 2025071914* (HTC); ibid., *W.-Z. Huang et al. 2025071915* (HTC); ibid., *W.-Z. Huang et al. 2025071906* (HTC); ibid., *W.-Z. Huang et al. 2025071916* (HTC); ibid., *W.-Z. Huang et al. 2025071904* (HTC); ibid., *W.-Z. Huang et al. 2025071909* (HTC); ibid., *W.-Z. Huang et al. 2025071912* (HTC); Altay City, Qiemuerqieke Town, along Ahe Highway (G681), on soil, 48°17′49.79″ N, 88°18′25.32″ E, 2553 m, 20 July 2025, *W.-Z. Huang et al. 20250720-13* (HTC); ibid., W.-Z. *Huang et al. 20250720-17* (HTC); Altay City, Altai Mountains, Wuqilike National Wetland Park, on a soil slope, 48°02′44.62″ N, 88°19′27.99″ E, 2100 m, 5 July 2016, *M. Sulayman 27879* (XJU); Burqin County, Altai Mountains, Heihu Lake, Kanas National Nature Reserve, on soil, 48°41′16.42″ N, 87°14′17.70″ E, 2190 m, 10 July 2016, *M. Sulayman 28095* (XJU); Fuhai County, Altai Mountains, Tiesikedala, on soil, 47°59′51.68″ N, 88°29′33.87″ E, 2552 m, 19 July 2022, *M. Sulayman 25954* (XJU); Ili Kazak Autonomous Prefecture, Nileke County, along Duku Highway (G217), near the entrance to Hashilegen Tunnel, on soil and rock, 43°43′45.23″ N, 84°25′02.98″ E, 3462 m, 27 July 2025, *W.-Z. Huang et al. 2025072728* (HTC); ibid., *W.-Z. Huang et al. 2025072736* (HTC); ibid., *W.-Z. Huang et al. 2025072726* (HTC). Xizang: Linzhi City, Bomi County, along the old road near the entrance to Galongla Tunnel, Bomi–Motuo Highway (G559), on soil, 29°45′57.26″ N, 95°41′44.92″ E, 3861 m, 26 September 2025, *W.-Z. Huang 20250926-108* (HTC); Rikaze City, Gyirong County, Sale Township, near a mountain pass, on humus in shrubland, 28°22′57.81″ N, 85°24′51.00″ E, 4055 m, 15 July 2025, *L. Zhang 16089* (SZG).

***Bartramia laevisphaera***

**CHINA. Qinghai:** Guoluo Zangzu Autonomous Prefecture, Banma County, Dengta Township, Mazigou, on soil, 32°40′56.39″ N, 100°59′41.62″ E, 3331 m, 25 August 2025, *W.-Z. Huang 20250825-18* (HTC). **Xizang:** Linzhi City, Lulang County, Dongga Village, on soil. 29°02′06.00″ N, 93°09′31.00″ E, 3100 m. 15 July 2020, *F.-H. Li 20200715P2G1012* (XZ).

***Bartramia pomiformis***

**CHINA. Guizhou:** Tongren City, Fanjingshan Mountain, near copper mine, on thin soil over rock surface, 800 m, 22 October 1979, *Z.-Y. Li 00074* (IFP 15867). **Heilongjiang:** Haerbin City, Wuchang City, Fenghuangshan National Forest Park, on stone, 128°4′16.05″ E, 44°11′53.76″ N, 922 m, 28 August 2025, *C.-F. Chen 2096* (LBG). **Hubei:** Enshi Tujia and Miao Autonomous Prefecture, Xingdoushan Mountain, on rock, 940 m, 26 October 2005, *X.-Q. Wang 7466* (HGTC); Xingdoushan Mountain, on soil, 820 m, 28 November 2005, *X.-Q. Wang 7559* (HGTC); Xiangyang City, Baokang County, Houping Town, Fenshuiling Village, on soil, 31°44′25″ N, 111°06′18″ E, 1168 m, 31 May 2025, *W.-Z. Ma 15977* (KUN); Baokang County, Longping Town, Wudaoxia National Nature Reserve, Lijiachong, on soil, 31°42′27″ N, 111°24′55″ E, 1400 m, 20 October 2025, *W.-Z. Ma et al. 17171* (KUN). **Inner Mongolia**: Hulunbuir City, Genhe City, Da Hinggan Ling, Genheyuan National Wetland Park, on a roadside rocky slope and at the base of a tree, 50°56′25.50″ N, 121°49′16.53″ E, 773 m, 2 August 2022, *R.-L. Zhu et al. 20220802-41* (HSNU); ibid., *R.-L. Zhu et al. 20220802-44* (HSNU). **Jiangxi:** Shangrao City, Yushan County, Sanqingshan Mountain, Jinsha, on soil, 410 m, 13 September 1985, *E.-Z. Bai & J.-Y. Feng 37197* (IFP). **Jilin:** Baishan City, Linjiang City, Huashan Town, Houdawozi, Jilin Ganfanpen National Forest Park, on soil over a rock wall, 41°55′10.07″ N, 126°46′27.29″ E, 6 m, 3 May 2026, *M.-Y. Hu et al. 2026050301* (HTC). **Qinghai:** Guoluo Zangzu Autonomous Prefecture, Banma County, Maheke Township, along X709 Road, on soil, 33°05′03.60″ N, 100°25′21.14″ E, 3836 m, 23 August 2025, *W.-Z. Huang 20250823-5* (HTC). **Zhejiang:** Hangzhou City, Chun’an County, Jinzijian, on rock, 29°47′52.52″ N, 118°57′55.84″ E, 710 m, 27 August 2020, *Y.-H. Wu & J.-M. Shen 2020082731* (HTC); Lin’an District, Qingliangfeng Town, Baiguo Village, Daming Mountain, on wet rock at mine entrance, 30°02′02.92″ N, 118°58′59.76″ E, 1089 m, 6 July 2018, *J.-X. Guo et al. 20180706-100* (HTC); Lin’an District, Qingliangfeng Town, Baiguo Village, Daming Mountain, on soil over a rock wall, 30°02′11.80″ N, 118°58′45.30″ E, 1208 m, 8 June 2026, *W.-Z. Huang & Y.-S. Liu 20260608-9* (HTC); ibid., 30°02′11.20″ N, 118°58′45.44″ E, 1140 m, *W.-Z. Huang & Y.-S. Liu 20260608-15* (HTC); ibid., 30°02′23.55″ N, 118°58′53.20″ E, 854.99 m, *W.-Z. Huang & Y.-S. Liu 20260608-3* (HTC); ibid., 30°02′11.80″ N, 118°58′45.30″ E, 1108.49 m, *W.-Z. Huang & Y.-S. Liu 20260608-12* (HTC); Huzhou City, Anji County, Anji Hynobiid National Nature Reserve, on a rock wall, 30°24′29.35″ N, 119°26′09.53″ E, 974 m, 3 September 2020, *W.-Z. Huang et al. 20200903045* (HTC); Hangzhou City, Lin’an District, Qingliangfeng Town, Shimenxia Longmen Scenic Area, on rock beside water, 30°08′35″ N, 118°51′54″ E, 677 m, 29 August 2026, *R.-L. Zhu et al. 20260829-220* (HSNU); Anji County, Anji Xianhai Villa, on a rock wall beside a gully, 30°29′02.90″ N, 119°38′43.95″ E, 237 m, 3 October 2022, *W.-Z. Huang et al. 2022100336* (HTC); Anji County, Longwangshan Nature Reserve, Qianmutian, on soil, 18 April 2013, *Y.-H. Wu 2013041818* (HTC); Anji County, Zhangcun Town, Longwangshan Scenic Area at the source of the Huangpu River (Mafeng’an–Xiguan), on roadside rocks, 30°23′28.14″ N, 119°24′43.17″ E, 819 m, 2 May 2021, *X.-Y. Ma et al. 2021050220* (HTC); Anji County, Shilongtou Mountain, on a rock wall at the mountain summit, 30°25′14.55″ N, 119°25′19.28″ E, 784 m, 30 April 2021, *X.-Y. Ma & J.-H. Wang 2021043024* (HTC); ibid., on a rock wall in forest, 30°25′14.19″ N, 119°25′18.38″ E, 780 m, 30 April 2021, *X.-Y. Ma & J.-H. Wang 2021043026* (HTC); Anji County, Tongjiachang Natural Village, Xiaoyaogu Homestay, on soil over a rock wall beside a stream/gully, 30°25′13.10″ N, 119°31′18.09″ E, 767 m, 5 May 2021, *X.-Y. Ma et al. 2021050501* (HTC); Jinhua City, Yongkang City, Xiangzhu Town, Tanglikeng, on a soil slope, 30°40′06.22″ N, 119°50′23.39″ E, 265 m, 28 January 2024, *Y.-T. Xiang et al. 2024012826* (HTC); Lishui City, Liandu District, Fengyuan Township, on soil, 28°11′04.26″ N, 119°48′16.31″ E, 1132.44 m, 21 March 2025, *W.-Z. Huang et al. 20250321-17* (HTC); Liandu District, Fengyuan Township, on soil, 28°12′23.6268″ N, 119°49′45.7716″ E, 1022 m, 22 March 2025, *W.-Z. Huang et al. 20250322-11* (HTC); ibid., on soil, 28°11′04.26″ N, 119°48′16.31″ E, 1132.44 m, 26 March 2025, *W.-Z. Huang et al. 20250326-17* (HTC); Longquan City, Fengyangshan Nature Reserve, along the road from Peony Garden Leisure Area to Juebi Qisong, on soil, 27°54′04.53″ N, 119°11′41.28″ E, 1277 m, 28 August 2023, *X.-Y. Ma & J. Su 20230828125* (HTC); Qingyuan County, Xianliang Town, toward Fulin Village, on soil, 27°37′53.41″ N, 119°13′47.74″ E, 958 m, 31 December 2022, *X.-Y. Ma & W.-Q. Huang 2022123134* (HTC); Qingyuan County, from Gangtou Village toward Shimo Xia Village bridge, on a roadside rock wall, 27°33′49.43″ N, 119°19′23.61″ E, 676 m, 25 August 2022, *Y.-T. Xiang & W.-H. Zeng a2022082568* (HTC); Songyang County, Sidu Township, along the route from Shili Yanbei Forest Farm to Xiabao Village, on thin soil over a rock wall, 28°30′18.82″ N, 119°32′07.73″ E, 627 m, 19 July 2023, *X.-Y. Ma et al. 2023071971* (HTC); Songyang County, through primary forest to Shirenji, on rock, 28°18′26.83″ N, 119°16′27.12″ E, 1199 m, 5 August 2017, *W. Sheng et al. 20170805019* (HTC); Suichang County, Suzhouling, Jiulongshan Nature Reserve, on rock, 28°17′29.38″ N, 118°39′55.44″ E, 639 m, 20 July 2019, *W. Sheng et al. 2019072028* (HTC); Suichang County, along the road from Jiulong ticket office, on rock, 28°22′39.38″ N, 118°51′07.47″ E, 559 m, 12 July 2022, *X.-Y. Ma et al. 2022071241* (HTC); Suichang County, proposed expansion area of Jiulongshan Nature Reserve, along the route from Zuobieyuan Power Station to Yangmeikeng, on rock, 28°18′00.65″ N, 118°45′49.85″ E, 571 m, 22 July 2023, *Y.-T. Xiang et al. 2023072219* (HTC); Yunhe County, Chongtou Town, Qixingdun Scenic Area, Yunhe Terraces, ancient trail from Yunman Homestay to Meijiujian, on a moist rock wall, 28°01′23.76″ N, 119°27′01.85″ E, 1096 m, 10 July 2023, *X.-Y. Ma et al. 2023071028* (HTC); Quzhou City, Jiangshan City, Nianbadu Town, Fugai Mountain National Forest Park, on rock, 28°15′08.36″ N, 118°28′39.44″ E, 680 m, 21 April 2017, *W. Sheng et al. 2017042153-1* (HTC); Shimen Town, Jianglangshan Scenic Area, on rock, 28°31′50.44″ N, 118°33′47.72″ E, 473 m, 17 August 2017, *W. Sheng et al. 20170817022* (HTC); Bao’an Township, Xianxialing Nature Reserve, on rock, 28°20′57.98″ N, 118°37′55.68″ E, 589 m, 25 January 2019, *W. Sheng & W.-Z. Huang 2019012562* (HTC); Wenzhou City, Cangnan County, Juxi Town, on thin soil over rock surface, 27°31′51.92″ N, 120°07′18.86″ E, 544 m, 13 April 2018, *W.-Z. Huang et al. 2018041313* (HTC).

***Bartramia subulata***

**CHINA. Qinghai:** Guoluo Zangzu Autonomous Prefecture, Banma County, Jika Township, along Provincial Road 709, on soil in a meadow, 32°55′16.25″ N, 100°18′33.66″ E, 4510 m, 23 August 2025, *W.-Z. Huang 20250823-7* (HTC). **Sichuan:** Aba Tibetan and Qiang Autonomous Prefecture, Xiaojin County, Balangshan Pass, on soil, 30°53′46.31″ N, 102°54′33.05″ E, 3982 m, 30 June 2026, *W.-Z. Huang 20260630-6* (HTC). **Xizang:** Rikaze City, Yadong County, on soil, 27°36′42.20″ N, 88°50′15.97″ E, 4395 m, 20 September 2020, *Y.-L. Xiang et al. 20200920-42B* (HSNU).
